# Exceptional n-type thermoelectric ionogels enabled by metal coordination and ion-selective association

**DOI:** 10.1126/sciadv.adk2098

**Published:** 2023-10-25

**Authors:** Wei Zhao, Yiwei Zheng, Meng Jiang, Tingting Sun, Aibin Huang, Lianjun Wang, Wan Jiang, Qihao Zhang

**Affiliations:** ^1^ State Key Laboratory for Modification of Chemical Fibers and Polymer Materials, College of Materials Science and Engineering, Donghua University, Shanghai 201620, China.; ^2^ Soochow Institute for Energy and Materials Innovations, College of Energy, Key Laboratory of Advanced Carbon Materials and Wearable Energy Technologies of Jiangsu Province, Soochow University, Suzhou 215006, China.; ^3^ State Key Laboratory of High Performance Ceramics and Superfine Microstructure, Shanghai Institute of Ceramics, Chinese Academy of Sciences, Shanghai 200050, China.; ^4^ Center of Materials Science and Optoelectronics Engineering, University of Chinese Academy of Sciences, Beijing 100049, China.; ^5^ Institute of Functional Materials, Donghua University, Shanghai 201620, China.; ^6^ Institute for Metallic Materials, Leibniz Institute for Solid State and Materials Research, Dresden 01069, Germany.

## Abstract

Ionic liquid–based ionogels emerge as promising candidates for efficient ionic thermoelectric conversion due to their quasi-solid state, giant thermopower, high flexibility, and good stability. P-type ionogels have shown impressive performance; however, the development of n-type ionogels lags behind. Here, an n-type ionogel consisting of polyethylene oxide (PEO), lithium salt, and ionic liquid is developed. Strong coordination of lithium ion with ether oxygen and the anion-rich clusters generated by ion-preferential association promote rapid transport of the anions and boost Eastman entropy change, resulting in a huge negative ionic Seebeck coefficient (−15 millivolts per kelvin) and a high electrical conductivity (1.86 millisiemens per centimeter) at 50% relative humidity. Moreover, dynamic and reversible interactions among the ternary mixtures endow the ionogel with fast autonomous self-healing capability and green recyclability. All PEO-based ionic thermoelectric modules are fabricated, which exhibits outstanding thermal responses (−80 millivolts per kelvin for three p-n pairs), demonstrating great potential for low-grade energy harvesting and ultrasensitive thermal sensing.

## Teaser

Self-healing n-type PEO ionogels for efficient ionic thermoelectric conversion are developed.

## INTRODUCTION

Ionic thermoelectric (iTE) materials driven by the Soret effect are receiving increasing interest in areas such as energy harvesting from low-grade heat and high-sensitivity sensors due to their giant thermopower (*1*–*3*). The ionic Seebeck coefficient (*S*_i_) can reach an order of magnitude of 10 mV K^−1^ arising from thermal diffusion of ions under temperature gradients (*4*), which offers advantages of avoiding the use of a large number of thermoelements to magnify the signal output and producing more sensitive sensors compared to electronic thermoelectric materials (*5*–*7*).

Various iTE materials, including polyelectrolytes, polyelectrolytes in conductive polymers, ionic liquid (IL)–based ionogels, and hybrid materials, have been successfully designed and developed (*4*). Among them, IL-based ionogels stand out because of their quasi-solid state, high flexibility, nonvolatility, good thermal stability, low cost, and scalable processability. They typically consist of a solid polymer matrix that provides material strength and an IL that acts as a dispersion medium and source of ions (*8*). In general, if cations dominate thermal diffusion, then the IL-based ionogels have p-type conductive behavior, with *S*_i_ being positive, and vice versa for n-type (*9*). Over the past few years, a range of strategies have been proposed to enhance *S*_i_ and/or ionic electrical conductivity (σ_i_) of IL-based ionogels, such as tailoring the interaction between polymers and ions (*10*, *11*), adding inorganic fillers (*12*), ion doping (*13*), and electrode design (*14*). As a result, thermoelectric ionogels with stunning thermoelectric properties have been reported. For instance, Liu *et al.* (*13*) achieved a giant *S*_i_ of 43.8 mV K^−1^ with a high σ_i_ of 19.4 mS cm^−1^ in poly(vinylidene fluoride-*co*-hexafluoropropylene) (PVDF-HFP)/1-ethyl-3-methyl imidazolium dicyanamide (EmimDCA)/sodium dicyanamide (NaDCA) at a relative humidity (RH) of 85% and room temperature.

Ionic thermopiles are typically composed of both p-type and n-type iTE materials connected in electrical series and thermal parallel to generate sufficient energy. However, despite notable advances in achieving giant ionic properties, most state-of-the-art IL-based thermoelectric ionogels are p-type materials. In contrast, n-type ionogels with satisfactory negative *S*_i_ and high σ_i_ have scarcely been reported (*8*, *9*). A pioneering work by Zhao *et al.* (*10*) presented an “ambipolar” thermoelectric ionogel by using PVDF-HFP, Emim bistrifluoromethyl sulfony imide (EmimTFSI), and poly(ethylene glycol). The sign of *S*_i_ was successfully shifted from positive (+14 mV K^−1^) to negative values (−4 mV K^−1^). Recently, Liu *et al.* (*15*) adopted the ion-ion interactions to selectively modulate the sign and magnitude of *S*_i_ for PVDF-HFP/EmimTFSI. They achieved impressive tuning within the range from +17 to −15 mV K^−1^ at an RH of 60% and near room temperature. Despite these breakthroughs, research on n-type ionogels is still in its infancy, and the material diversity and their properties are still inferior to those of p-type (*16*). Thus, to date, the lack of high thermoelectric performance and reproducible n-type ionogels still hinders the implementation of ionic thermopiles in practical applications, and it remains challenging to modulate the interactions between the polymer matrix and the ions to obtain extraordinary n-type thermoelectric ionogels.

In addition to excellent iTE properties, ionogels with fascinating mechanical properties, such as sufficient stretchability and spontaneous self-repairing ability, are also highly desired to meet the demands of wearability and motion applications such as biosensors, electronic skins, and smart clothes (*17*). This is because wearable devices are vulnerable to mechanical deformation such as bending, flexing, and even accidental scratches or cuts. The introduction of stretchability and self-healing properties can substantially resist unexpected mechanical damage and prevent permanent breakdowns, thus greatly extending the longevity of a device and reducing maintenance costs. In a previous study, Xu *et al.* (*17*) designed a stretchable and self-healable ionogel with p-type iTE properties using polyurethane, EmimDCA IL, and a kind of dioxaborolane cross-linker. The material showed a mechanical stretchability of 300%, an *S*_i_ of +15.6 mV K^−1^, and a σ_i_ of 2.1 mS cm^−1^ at an RH of 50%, as well as rapid self-healing ability in the absence of any external stimuli. With regard to n-type ionogels, a PVDF-HFP–based iTE material was recently demonstrated (*16*). Although stretchable and self-healable ionogels with negative *S*_i_ (−11.5 mV K^−1^ at an RH of 50%) were obtained, the corresponding σ_i_ was as low as 3 × 10^−2^ mS cm^−1^. To date, there have been few n-type ionogels that are capable of achieving prominent *S*_i_ and high σ_i_ while also featuring commendable mechanical properties such as stretchability and self-healing ability.

**Fig. 1.**
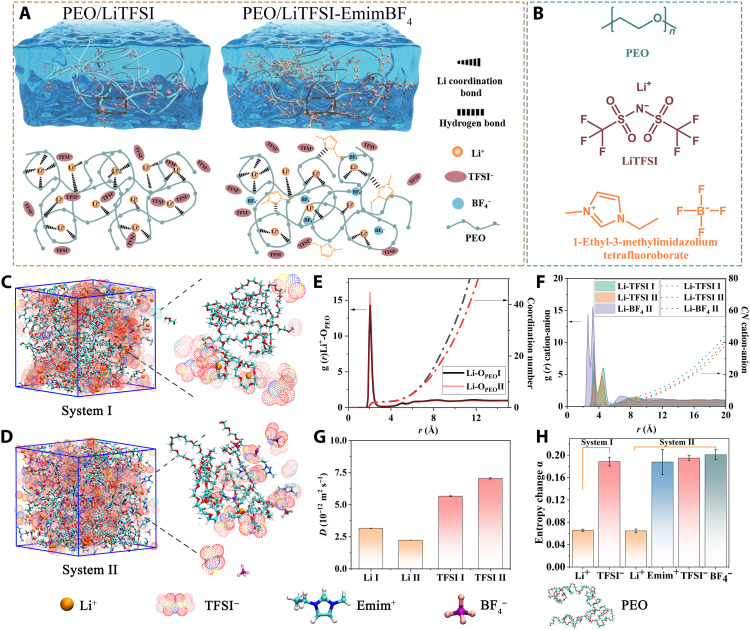
Design principle for n-type PEO-based thermoelectric ionogels with metal coordination and ion-selective association. (**A**) Schematic illustration of ionogels (PEO/LiTFSI and PEO/LiTFSI-EmimBF_4_) showing the interaction between polymer (PEO), lithium salt (LiTFSI), and IL (EmimBF_4_). The combination of PEO and LiTFSI (system I) forms an ionogel with metal-ligand coordination and ion association. The combination of PEO, LiTFSI, and EmimBF_4_ (system II) forms a more complex network featuring metal-ligand coordination, ion association, and hydrogen bonding. (**B**) Chemical structures of PEO, LiTFSI, and EmimBF_4_. (**C** and **D**) MD snapshots of PEO/LiTFSI and PEO/LiTFSI-EmimBF_4_, respectively. The atomic structures of PEO, LiTFSI, and EmimBF_4_ are shown at the bottom of this figure. (**E** and **F**) RDFs and CNs of Li^+^-O_PEO_ and Li^+^-TFSI^−^, Li^+^-BF_4_^−^, respectively. (**G** and **H**) Diffusion coefficient and entropy change α calculated from MD simulations.

Here, we report a polyethylene oxide (PEO)–based n-type ionogel (Fig. 1, A and B), which consists of a PEO polymer matrix, a lithium salt [lithium bis(trifluoromethanesulfonyl)imide (LiTFSI)], and an IL [Emim tetrafluoroborate (EmimBF_4_)]. A strategy using metal-ligand coordination and ion-selective association is proposed to boost the anion transport and increase the transfer entropy. Molecular dynamics (MD) simulations reveal that the presence of anion clusters and preferential ion association further facilitates anion diffusion. As a result, superior iTE performance is achieved with an *S*_i_ of −15 mV K^−1^ and a σ_i_ of 1.86 mS cm^−1^ at an RH of 50%. Furthermore, this ionogel is not only flexible and stretchable because of the soft nature of the polymer complex but also self-healing rapidly without any external stimuli, which stems from the dynamic and reversible physical cross-linking between lithium ions and ether oxygen of PEO chains. Beyond these, because of the reversible physical entanglement based on noncovalent interactions, the ionogel can be dispersed in ethanol and regenerated by recoordination upon evaporation of the solvent, leading to green recovery and recycling, which have rarely been achieved in other reported ionogels.

## RESULTS

### Design n-type thermoelectric ionogels

Theoretically, the ionic Seebeck coefficient can be given as follows (*1*, *9*):

$$S_{i}=\frac{\sum_{i}q_{i}n_{i}^{0}\hat{S_{i}}D_{i}}{\sum_{i}q_{i}^{2}n_{i}^{0}D_{i}}$$

where *i*, *q*, *n*^0^, $\hat{S}$, and *D* represent ion species, charges, ion concentration, the Eastman entropy, and diffusion coefficient, respectively. Obviously, the sign and magnitude of the ionic Seebeck coefficient are related to the Eastman entropy and the migration difference between cations and anions. As reported in previous work, the Eastman entropy is related to the interactions between ions and surrounding environment, with both strong ion-ion interactions and ion-dipole interactions leading to high entropy and thus higher Seebeck coefficients (*18*, *19*). The synergistic and competitive effects between ion-polymer and ion-ion interactions are essential characteristics in iTE gels. To obtain an n-type ionogel, we selected LiTFSI as the ionic source in this work because of its low cost, high stability, and weak coordination between cations and anions; at the same time, PEO was chosen as the polymer matrix, taking into account its ability to dissolve lithium salts, good mechanical stability, and low lithium ion transference number of less than 0.5 in their binary complex (*20*, *21*). The solvent-free ionogel containing PEO and LiTFSI is designated as PEO/LiTFSI. In this regime, the characteristic CH_2_─CH_2_─O repeating units could solvate LiTFSI through the strong coordination between Li^+^ with ether oxygens, yielding amounts of dissociated cations and anions as charge carriers (*22*). Since the TFSI^−^ anions are weakly coordinated to PEO, they are more free-moving than Li^+^ and migrate faster than Li^+^ (*23*), rendering the ionogel with n-type iTE performance. Moreover, considering the relative sluggish ion diffusion of the PEO-based electrolyte, EmimBF_4_ IL was further introduced. The corresponding ionogel is named PEO/LiTFSI-EmimBF_4_. By the introduction of abundant ion associations and ion-dipole interactions, the ionic conductivity and Seebeck coefficient are expected to be significantly improved.

To support the design rationale of this novel n-type PEO/LiTFSI-EmimBF_4_ ionogel, we first performed MD simulations to reveal the interaction mechanism by analyzing the microscopic structures and diffusion dynamics of ions in PEO/LiTFSI (system I) and PEO/LiTFSI-EmimBF_4_ (system II). The snapshot of system I demonstrates the existence of Li-TFSI clusters in PEO/LiTFSI at high concentration of LiTFSI (Fig. 1C) because the ether oxygen units in PEO can provide a negative charge environment to coordinate with Li^+^. These Li-TFSI clusters show asymmetry where anions are dominant, leading to a reduced contribution to the cooperative migration of Li^+^. After adding EmimBF_4_ to PEO/LiTFSI, Li-TFSI clusters are reduced, and more freely diffusing TFSI^−^ can be observed (Fig. 1D). The radial distribution function (RDF) and coordination number (CN) of these two systems were further calculated. In PEO/LiTFSI, both Li-O_TFSI_ (see fig. S1) and Li-O_PEO_ (Fig. 1E) show representative sharp peaks at 2.05 Å, indicating that Li^+^ cations are surrounded by both PEO chains and TFSI^−^ as the first coordination shell, and there is a competition from the ether oxygen and TFSI^−^. In addition, the higher amplitude of Li-O_TFSI_ than that of Li-O_PEO_ combined with the presence of bimodal peaks at 2.05 and 4.46 Å verifies the simultaneous formation of Li-TFSI clusters (*22*). From the analysis of CNs in Fig. 1E, there is an average of 5.417 oxygen atoms surrounding each Li^+^ in PEO/LiTFSI, of which 3.156 oxygen atoms come from TFSI^−^ and the other from PEO (see table S1). The total CN of Li-O and the asymmetric clustering phenomena are consistent with previous MD simulation studies on the ion association properties of PEO-LiTFSI electrolytes (*22*, *24*). In contrast, the peaks of Li-O_TFSI_ are weakened in the PEO/LiTFSI-EmimBF_4_ ternary system (see fig. S1). The CN of Li-O_TFSI_ decreases sharply from 3.156 to 1.703, while CN of Li-O_PEO_ is almost unchanged (see table S1). These results indicate that the introduction of EmimBF_4_ has minimal effect on the coordination of Li^+^ and PEO chains. Alternatively, the ion cluster structure has been changed because of ion association, releasing more TFSI^−^. The RDFs and CNs for the Li^+^-TFSI^−^ and Li^+^-BF_4_^−^ disclose the main reason of the structural change. As shown in Fig. 1F, the first peak position of Li^+^-BF_4_^−^ is located at 2.625 Å, which is smaller than that of Li^+^-TFSI^−^. In addition, Li^+^-BF_4_^−^ shows higher peak intensity and more CN. These results suggest that Li^+^-BF_4_^−^ clusters can be more tightly associated because of stronger electrostatic interactions.

As mentioned above, the ion-polymer coordination and ion-ion association have a significant effect on the migration of ions, which play a decisive role in determining the sign and magnitude of ionic Seebeck coefficient. Hence, we further calculated the ion self-diffusion coefficients *D* and the entropy change α. The *D* values are calculated from the ionic mean square displacement (MSD) curves (see fig. S2) through a three-dimensional diffusion relationship: ${\mathrm{MSD}}_{\mathrm{ion}}(t)\equiv {\underset{{r(t)}_{\mathrm{ion}}}{\to }}^{2}=6D_{\mathrm{ion}}t$ (*23*). The corresponding *D* values of Li^+^ and TFSI^−^ are shown in Fig. 1G. It can be seen that *D*_TFSI_^−^ is larger than *D*_Li_^+^ in both PEO/LiTFSI and PEO/LiTFSI-EmimBF_4_, implying that they will be n-type conducting ionogels. Moreover, *D*_Li_^+^ decreases, while *D*_TFSI_^−^ increases in PEO/LiTFSI-EmimBF_4_, which is consistent with the release of freer TFSI^−^ after adding EmimBF_4_. The enlarged migration difference between cations and anions will result in the enhancement of ionic Seebeck coefficient. The temperature-dependent entropy values of cation and anion in PEO/LiTFSI and PEO/LiTFSI-EmimBF_4_ are shown in fig. S3. The statistical entropies of ions present a linear relationship with temperature, where the entropy change α is obtained from the slope of the lines. As shown in Fig. 1H, α of TFSI^−^ (0.1885) is larger than that of Li^+^ (0.0658) in PEO/LiTFSI. There is a clear trend of $\alpha_{{\mathrm{BF}}_{4}^{-}}$ > α_TFSI^−^_ > α_Emim^+^_ > α_Li^+^_. According to the equation above, anions with larger entropy dominate the contribution to ionic Seebeck coefficient. Moreover, the larger entropy change of anions means the greater difference of corresponding entropy at given temperature gradient, and, thus, the larger *S_i_* will be obtained. In all, both diffusion coefficient values and entropy change of anions are larger than those of cations in our designed PEO-based ionogels, indicating that anions have a stronger driving force and thus will contribute to n-type ionogels high ionic Seebeck coefficients and conductivities (*9*).

### Structural and thermoelectric characterization

**Fig. 2.**
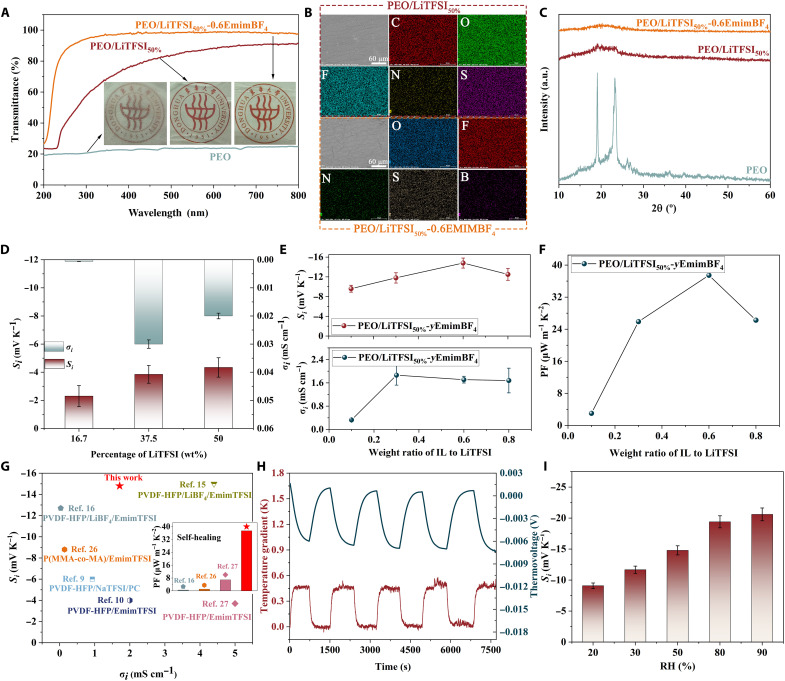
Effect of composition on transmittance, crystallinity, and thermoelectric properties. (**A**) Ultraviolet-visible transmittance spectra. Insets are the corresponding photos with a scale bar of 1 cm. (**B**) Elemental mapping results. Scale bars, 60 μm. (**C**) XRD patterns. a.u., arbitrary units. (**D** and **E**) Ionic Seebeck coefficient (*S*_i_) and ionic electrical conductivity (σ_i_) of PEO/LiTFSI and PEO/LiTFSI_50%_-EmimBF_4_ ionogels, respectively. (**F**) Power factors of PEO/LiTFSI_50%_-EmimBF_4_ ionogels. (**G**) Comparison of *S*_i_ and σ_i_ of PEO/LiTFSI_50%_-0.6EmimBF_4_ ionogel in this work with previously reported n-type ionogels (*9*, *10*, *15*, *16*, *26*, *27*). Data at the RH of 50 or 60% are taken. Inset compares the power factors of n-type ionogels with self-healing ability. (**H**) Thermovoltage and temperature curves of PEO/LiTFSI_50%_-0.6EmimBF_4_ ionogel under several continuous cycles. (**I**) *S*_i_ of PEO/LiTFSI_50%_-0.6EmimBF_4_ ionogel at different relative humidities.

Experimentally, we first synthesized binary PEO/LiTFSI*_x_* ionogels (see Materials and Methods). Here, *x* is the weight percentage of LiTFSI relative to the total weight of PEO and LiTFSI. Then, ternary ionogels in composition of PEO/LiTFSI_50%_-*y*EmimBF_4_ were synthesized, where PEO/LiTFSI_50%_ was used as a reference sample and *y* denotes the weight ratio of EmimBF_4_ to LiTFSI. As a result, we can see from the appearance of the samples that PEO/LiTFSI_50%_ and PEO/LiTFSI_50%_-0.6EmimBF_4_ present a higher degree of transparency compared to PEO (Fig. 2A). This is due to the good compatibility among PEO, LiTFSI, and EmimBF_4_. The transmittance of PEO/LiTFSI_50%_-0.6EmimBF_4_ exceeds 98% at wavelengths of 300 to 800 nm. The elemental mapping results indicate the homogenous distribution of C, O, F, N, S, and B elements in PEO/LiTFSI_50%_ and PEO/LiTFSI_50%_-0.6EmimBF_4_ ionogels (Fig. 2B), which confirms that LiTFSI and EmimBF_4_ are well dispersed in PEO matrix. X-ray diffraction (XRD) results (Fig. 2C) show that PEO has two distinct diffraction peaks corresponding to its inherent high crystallinity, which usually hinders ionic transport and results in a rather low ionic conductivity. In contrast, after the introduction of LiTFSI and EmimBF_4_, the sharp diffraction peaks diminish, and the degree of crystallinity is markedly suppressed. This can facilitate ion migration in the amorphous phase (*25*).

Further, we examined the thermoelectric properties of the samples. As expected, the binary and ternary ionogels both show n-type conductive properties (Fig. 2, D and E), which is consistent with our design concept. Specifically, *S*_i_ increases with increasing LiTFSI content, from −2.3 mV K^−1^ for PEO/LiTFSI_16.7%_ to −4.3 mV K^−1^ for PEO/LiTFSI_50%_. However, when too much lithium salt (more than 50%) was added, the formability of the samples got deteriorated, and, thus, we did not optimize the thermoelectric properties by adding more lithium salt. At the same time, σ_i_ is substantially improved, from 0.00059 mS cm^−1^ for PEO/LiTFSI_16.7%_ to 0.033 mS cm^−1^ for PEO/LiTFSI_37.5%_, due to the increase in carrier concentration. However, a further increase of lithium salt content leads to a slight decrease in σ_i_ due to the ion aggregation at high concentrations. As for the ternary ionogels, *S*_i_ reaches an impressive value of −15 mV K^−1^ when the *y* value of EmimBF_4_ increases to 0.6 (Fig. 2E and fig. S4). This is attributed to the synergistic effect of metal coordination and ion-selective association, where the formation of strongly bound Li^+^, more freely moving TFSI^−^, and Li^+^-BF_4_^−^ clusters enables higher diffusion coefficients and entropy changes of the anions. The *S*_i_ of PEO/LiTFSI_50%_-0.8EmimBF_4_ decreases slightly to −12.5 mV K^−1^, which may be due to the migration of Emim^+^ when EmimBF_4_ content is higher. The ionic conductivities of PEO/LiTFSI_50%_-*y*EmimBF_4_ are evaluated by AC impedance spectroscopy (see fig. S5). As a result, σ_i_ is also enhanced, reaching a maximum value of 1.86 mS cm^−1^ for PEO/LiTFSI_50%_-0.3EmimBF_4_ (Fig. 2E). The calculated power factors (PFs) are shown in Fig. 2F. It can be seen that PFs increase firstly because of the simultaneous enhancement of the ionic Seebeck coefficient and conductivity. The maximum PF of 37.5 μW m^−1^ K^−2^ is obtained for PEO/LiTFSI_50%_-0.6EmimBF_4_. Compared with existing reports (*9*, *10*, *15*, *16*, *26*, *27*), our ionic Seebeck coefficient is comparable to the highest value currently available for n-type ionogels (Fig. 2G). Our PEO-based ionogels are self-healing (see subsequent discussion), and a stretchable thermoelectric ionogel with such a high PF is unprecedented (inset of Fig. 2G).

In addition, when PEO/LiTFSI_50%_-0.6EmimBF_4_ is subjected to a small temperature difference of 0.45 K, the thermovoltage is generated rapidly and remains unchanged during the repeated heat-on and heat-off cycles (Fig. 2H). This indicates that the ionogel has a sensitive and stable temperature response. The effect of humidity on PEO/LiTFSI_50%_-0.6EmimBF_4_ was further investigated. The measured samples were exposed to different relative humidities for 0.5 hours, followed by encapsulation with polyimide tapes. As a result, the *S*_i_ increases from −9.1 to −20.6 mV K^−1^ when RH increases from 20 to 90% (Fig. 2I). The σ_i_ also increases with increasing RH, and the maximum σ_i_ reaches 4.82 mS cm^−1^ at 90% RH (see fig. S6). Although the performance gets better at higher RH, we are more interested in the results at RH of 50 to 60% as this range is more suitable for human health and comfort (*25*). Moreover, only a slight reduction of *S*_i_ occurs after the ionogel was placed in a vacuum environment for 9 months, indicating excellent long-term stability (see fig. S7).

### Mechanisms of coordination and ion association

**Fig. 3.**
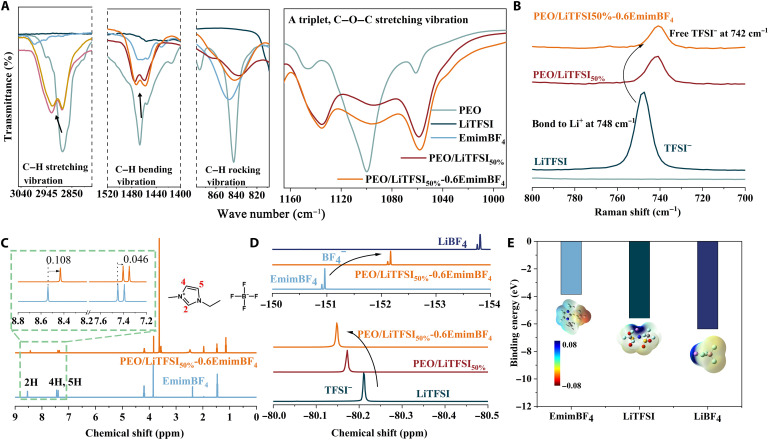
Interactions between ionogel components. (**A**) FTIR spectra of PEO, PEO/LiTFSI_50%_, and PEO/LiTFSI_50%_-0.6EmimBF_4_ show the stretching, rocking, and bending vibration band of C─H and stretching vibration band of C─O─C. (**B**) Raman spectra for TFSI^−^ in LiTFSI, PEO/LiTFSI_50%_, and PEO/LiTFSI_50%_-0.6EmimBF_4_ at range from 800 to 700 cm^−1^. (**C**) H-NMR spectra of EmimBF_4_ and PEO/LiTFSI_50%_-0.6EmimBF_4_ reveal the chemical shift of H on imidazolium cation. (**D**) F-NMR spectra to show the chemical shift of BF_4_^−^ and TFSI^−^. (**E**) Binding energy of EmimBF_4_, LiTFSI, and LiBF_4_. The inset shows the corresponding structure and electrostatic potential distribution.

From the above experimental results, the improvement of both ionic Seebeck coefficients and ionic conductivities of n-type PEO-based ionogels has been realized, implying that the introduction of LiTFSI and EmimBF_4_ plays a significant role in tuning of thermoelectric properties of PEO. To further elucidate the interactions present in the ionogel, detailed spectroscopic analyses were performed. The molecular interactions between the ions and PEO matrix were firstly analyzed using Fourier transform infrared (FTIR) spectroscopy. The bands in the regions of 2760 to 3060 cm^−1^, 1400 to 1520 cm^−1^, 808 to 880 cm^−1^, and 990 to 1165 cm^−1^ are attributed to the C─H stretching, bending, rocking, and the C─O─C stretching vibration modes (*28*), respectively (Fig. 3A and fig. S8A). We notice that the peaks of PEO matrix at 2883 and 1467 cm^−1^ split into two peaks in PEO/LiTFSI_50%_ and PEO/LiTFSI_50%_-0.6EmimBF_4_, and the intensity of the C─H bands decreases markedly. In addition, after the addition of LiTFSI to PEO, the two distinct bands at 962 and 947 cm^−1^ merge into a broader band and shift toward lower wave numbers (see fig. S8B). These changes in the band position, peak shape, and intensity of the C─H vibration modes imply that there is an interaction between Li^+^ and PEO chains. This is because the C─H vibrations are quite sensitive to the Li-O_PEO_ coordination, especially when the lithium salt content increases to a certain extent, one or more crystalline phase complexes with definite stoichiometric ratios are formed (*29*). The strong coordination between Li^+^ and ether oxygen of PEO makes the ions easier to dissociate, resulting in the formation of bonded cations and free anions and thus enlarging the ion migration difference. In addition, the band characteristics of PEO/LiTFSI_50%_ are almost consistent with that of PEO/LiTFSI_50%_-0.6EmimBF_4_, suggesting that the introduction of EmimBF_4_ has a negligible effect on the Li-O_PEO_ coordination. Moreover, the triple peaks near 1100 cm^−1^ that are attributed to the C─O─C stretching vibration are generally believed to be related to the crystallinity of PEO (*30*). Obviously, the coordination of Li^+^ with the ether oxygen also has a strong influence on this region, as the band at 1148 cm^−1^ shifts to 1135 cm^−1^ for PEO/LiTFSI_50%_ and 1134 cm^−1^ for PEO/LiTFSI_50%_-0.6EmimBF_4_, respectively. The shift to lower wave number is due to the conformational change of the PEO skeleton induced by alkali metal ions (*31*). Furthermore, the intensity of the strongest peak at 1104 cm^−1^ of PEO decreases after introducing LiTFSI, indicating that the crystallinity of PEO is suppressed, which is consistent with the above XRD results. In this context, the large anions in size act as plasticizers in PEO to increase the amorphous phases, which is favorable to ion migration.

Raman spectra further confirm the formation of free TFSI^−^ in PEO/LiTFSI_50%_ and PEO/LiTFSI_50%_-0.6EmimBF_4_ (Fig. 3B and fig. S9). The characteristic Raman band at 748 cm^−1^ for pure LiTFSI is assigned to the bonded Li^+^-TFSI^−^ ion pairs, whereas it shifts to 742 cm^−1^ after mixing with PEO and EmimBF_4_. This phenomenon differs from that recently reported in PVDF-HFP mixed with NaTFSI, which shows p-type ionic Seebeck coefficients (*9*). In this regard, we propose that the strong Li-O_PEO_ coordination is an essential prerequisite for the realization of high n-type ionic Seebeck coefficients in PEO-based ionogels. For further verification, we introduced NaTFSI and KTFSI, which feature weaker metal-coordination, into PEO matrix. It is found that the corresponding ionic Seebeck coefficients gradually decrease compared with the use of LiTFSI (see fig. S10A). In addition, as a counterexample, we adopted succinonitrile as an additive because it can facilitate the diffusion of Li^+^ by alleviating the affinity between Li^+^ and ester oxygen (*32*). As a result, the negative *S*_i_ decreases from −11.3 to −5.8 mV K^−1^ with increasing succinonitrile (see fig. S10B). These results support our view that the strong metal-ligand coordination and the weakly bound anions contribute to the formation of n-type ionogels.

In addition to studying the coordination between LiTFSI and PEO, the effect of EmimBF_4_ on the ion association was investigated by nuclear magnetic resonance (NMR) spectroscopy. Figure 3C shows the H-NMR spectra of EmimBF_4_ and PEO/LiTFSI_50%_-0.6EmimBF_4_. The signals of C^2^─H, C^4^─H/C^5^─H on Emim^+^ shift up by 0.108 and 0.046 parts per million (ppm), respectively, in PEO/LiTFSI_50%_-0.6EmimBF_4_. This upward shift implies the weakening effect of hydrogen-bonding interaction among Emim^+^ and BF_4_^−^ (*33*). Besides, ^19^F-NMR was used to confirm the existence of TFSI^−^ in PEO/LiTFSI_50%_ and additional BF_4_^−^ in PEO/LiTFSI_50%_-0.6EmimBF_4_. The fluorine signal of LiTFSI is located in −80.21 ppm (Fig. 3D). After mixing PEO with LiTFSI, a downward shift is observed, and a further downward shift occurs in PEO/LiTFSI_50%_-0.6EmimBF_4_. This indicates a gradual decrease in the presence of Li-TFSI ion pairs in the composites. On the contrary, the peak of BF_4_^−^ in the composites shifted upward compared to EmimBF_4_, which implies that Li^+^-BF_4_^−^ clusters formed by BF_4_^−^ increase (*34*, *35*). Ionic interactions account for the variation in the solvation structures. Meanwhile, density-functional theory calculations show that the binding energies of Emim^+^-BF_4_^−^, Li^+^-TFSI^−^, and Li^+^-BF_4_^−^ are −3.86, −5.77, and − 6.37 eV (Fig. 3E), respectively. Therefore, Li^+^ prefers to form ion pairs with BF_4_^−^ rather than TFSI^−^, which is consistent with the Raman and NMR results.

### Versatility of PEO-based ionogels

**Fig. 4.**
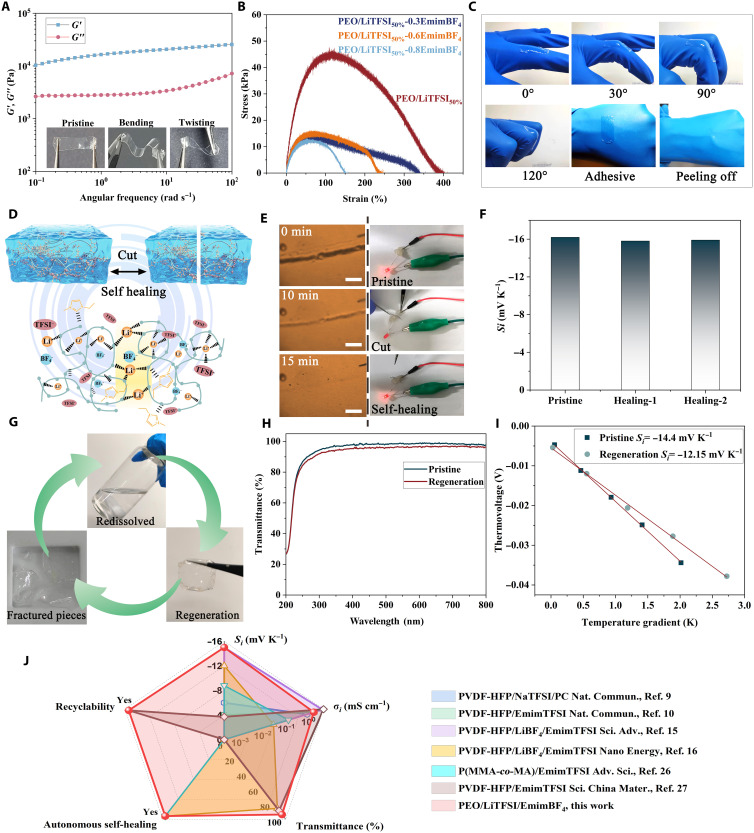
Versatility of PEO/LiTFSI_50%_-0.6EmimBF_4_ ionogel: Mechanical adaptability, self-healing, and recyclability. (**A**) Angular frequency dependence of storage modulus (*G′*) and loss modulus (*G*″). The insets are the photographs of PEO/LiTFSI_50%_-0.6EmimBF_4_ ionogel, showing its flexibility and twistability. (**B**) Tensile stress-strain curves at stretching rate of 10 mm min^−1^. (**C**) Photographs of a PEO/LiTFSI_50%_-0.6EmimBF_4_ ionogel attached onto a finger joint, demonstrating excellent adhesion, stretchability, and completely peeling from glove. (**D**) Schematic illustration of the self-healing mechanism. (**E**) Optical microscopy images of the self-healing process (scale bars, left, 500 μm) and demonstration of self-healing by connecting a light-emitting diode (right). (**F**) *S*_i_ of PEO/LiTFSI_50%_-0.6EmimBF_4_ before and after self-healing tests. (**G**) Flow diagram of the recyclability of PEO/LiTFSI_50%_-0.6EmimBF_4_ showing that the fractured ionogel pieces can be redissolved in ethanol and then regenerated. (**H**) Ultraviolet-visible transmittance spectra and (**I**) temperature gradient–dependent thermovoltage of the pristine and regenerated PEO/LiTFSI_50%_-0.6EmimBF_4_ ionogels. (**J**) Radar chart comparing the functionality of existing n-type ionogels.

The metal-ligand coordination and ion-ion association not only selectively improve the n-type iTE performance of PEO-based ionogels but also endow the ionogels with mechanical adaptability, self-healing, and recyclability. Dynamic mechanical measurements show that the storage modulus *G*′ of PEO/LiTFSI_50%_-0.6EmimBF_4_ is higher than the loss modulus *G*″ in the frequency range of 10^−1^ to 10^2^ rad s^−1^ (Fig. 4A), suggesting a quasi-solid gel behavior. The free-standing gel films exhibit good flexibility and bendability (inset in Fig. 4A), enabling intimate contact with heat source of different shapes. The stress-strain curve of the PEO-based ionogel in Fig. 4B and fig. S11 consists of three stages: linear viscoelasticity, damage accumulation, and failure states. After the linear viscoelastic state, the stress of the ionogel reaches a critical state, then microcracks appear, and the stress begins to deviate from the linear viscoelastic stress. The mechanical behavior of the ionogel eventually enters a failure state after reaching the maximum tensile strength, but the membrane does not completely rupture, so the strain is still increasing. The elongation at break of PEO/LiTFSI_50%_ is higher than that of PEO. However, the tensile strength decreases with the addition of LiTFSI and EmimBF_4_ due to their plasticizing effect. Higher EmimBF_4_ contents lead to higher swelling ratio and thus reduced mechanical strength and stretchability (*36*). In contrast to the highly chemically cross-linked gels with undesired stiffness, the PEO/LiTFSI_50%_-0.6EmimBF_4_ ionogel is soft because of dynamic and reversible coordination, which significantly improves the mechanical compliance and adhesion. As shown in Fig. 4C, the ionogel adheres conformably to the finger joint and accommodates finger movements, indicating desirable softness, stretchability, and good adhesion due to the limited polymer entanglements (*37*). The excellent mechanical adaptability prevents the ionogel from slipping or detaching from the substrate during movements, thus facilitating long-term stable temperature sensing or heat harvesting. In addition, the ionogel can be peeled off from objects (e.g., gloves) without residue, keeping the objects clean and free of contamination.

The PEO/LiTFSI_50%_-0.6EmimBF_4_ ionogel also exhibit excellent self-healing ability (Fig. 4D), which is achieved at room temperature without any external stimuli such as light or heat. Polarized optical microscopy is used to observe the healing process (Fig. 4E). The cuts heal immediately upon contact and almost completely disappear within 15 min, showing rapid spontaneous healing ability. This excellent self-healing property is further verified in a closed electrical circuit (Fig. 4E). A light-emitting diode bulb can be lit by connecting the circuit with a piece of PEO/LiTFSI_50%_-0.6EmimBF_4_ ionogel. When the gel in the circuit is cut in half, the bulb goes out; when the two cut-in-half gels are rejoined, the bulb relights immediately. In addition, after repeating the cut-heal tests, the ionic Seebeck coefficients remain more than 97% of that of the pristine ionogel (Fig. 4F and fig. S12). Tensile tests were also conducted on the cut and self-healed ionogels. Both the elongation at break and tensile strength remain unchanged after self-healing (see fig. S13). The outstanding self-healing capability is mainly related to the reversible Li^+^─O bonds and increased amorphous phase by TFSI^−^ as mentioned above. The metal-ligand coordination bonding at the broken interface can be reversibly reconstructed, and TFSI^−^ can effectively decrease the crystallinity and enhance the dynamics of the network, thus promoting the self-healing ability.

Moreover, because of the large number of reversible noncovalent intermolecular interactions between the linear PEO chains in PEO/LiTFSI_50%_-0.6EmimBF_4_, the ionogel can be dissolved in ethanol (Fig. 4G). This means that when the ethanol solvent molecules are removed by repouring and heating, the ionogel is reobtained by a rearranged network under the action of dynamic interactions. The regenerated ionogel also displays superior transmittance (Fig. 4H) and mechanical properties (see fig. S14) comparable to the pristine ionogel. In addition, the ionic Seebeck coefficients of the regenerated gels remain high (Fig. 4I), demonstrating good recyclability. This will provide an economical and practical strategy to guide the design of recyclable iTE materials and devices, paving the way for the development of electronic waste recycling.

Together, our PEO/LiTFSI_50%_-0.6EmimBF_4_ ionogel presents notable advantages over state-of-the-art n-type ionogels (Fig. 4J). The characteristics of high visible-light transmittance, superior ionic Seebeck coefficients, good ionic conductivities, fast autonomous self-healing ability, and environment-friendly recyclability make it a desirable candidate for sustainable thermal-electric conversion applications.

### All-PEO–based iTE devices

Thermoelectric ionogels can be used to directly charge capacitors or batteries to simultaneously generate and store electrical energy under intermittent heat sources (*25*); on the other hand, they can also be used as self-powered, highly sensitive thermal sensors to detect various kinds of heat (*10*). In both applications, higher output voltages are critical. For power generation, higher output voltages lead to higher output energy, while for sensors, higher output voltages yield larger output signals and higher detection resolution. To demonstrate the potential of iTE devices toward high voltages, we fabricated PEO-based iTE devices by connecting multiple thermoelectric legs electrically in series and thermally in parallel.

**Fig. 5.**
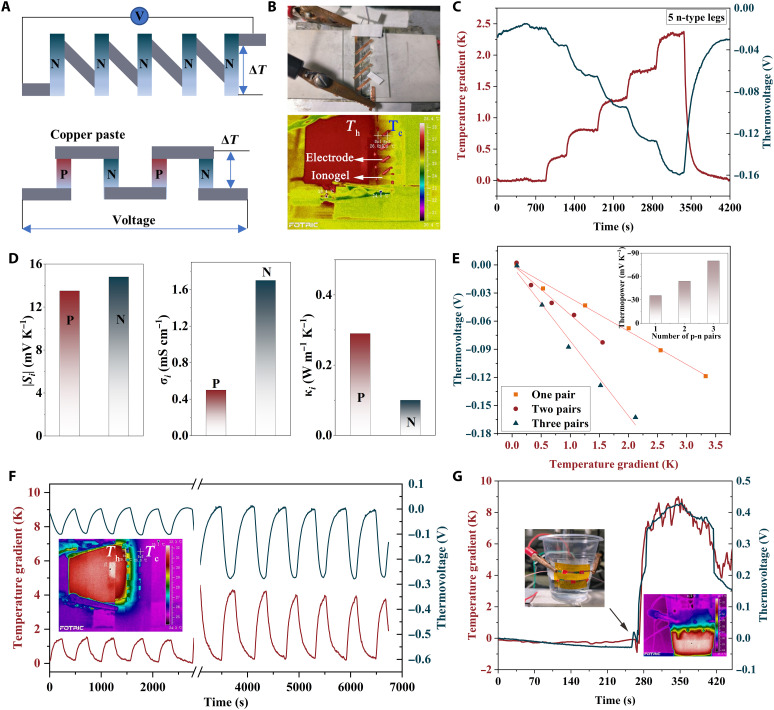
Preparation of all-PEO–based iTE devices and performance test. (**A**) Schematic illustration of iTE devices made of only n-type legs and p-n pairs. (**B**) Digital photograph and thermal infrared image of an n-type device with five legs. (**C**) Temperature gradient and corresponding thermovoltage of the n-type device. (**D**) iTE properties of the p/n PEO-based ionogels. (**E**) Generated thermovoltage of iTE devices with different number of p-n pairs. Inset is the corresponding thermopower. (**F**) Temperature gradient and corresponding thermovoltage of an iTE device containing two p-n pairs. (**G**) Demonstration of thermovoltage generated by two p-n pairs from a cup with warm water.

Two types of PEO-based devices are fabricated: n-type legs only and p-n pairs (Fig. 5A). Copper tapes were attached on a glass or polyimide substrate as the connecting electrodes, and then ionogels were drop casted. A device consisting of five n-type legs is assembled and shown in Fig. 5B and fig. S15. When a temperature difference is applied at both ends of the device, the thermal voltage shows a fast response and varies linearly with the increasing temperature gradient (Fig. 5C and fig. S16). The corresponding thermopower of the five legs reaches up to −63.4 mV K^−1^, confirming the potential of n-type PEO-based ionogels for low-grade thermal energy harvesting and high-sensitivity sensors. Nevertheless, the heat loss due to the electrodes result in the total thermopower of the five legs being less than the sum of the ionic Seebeck coefficients (ideally −75 mV K^−1^). In this regard, the use of p-n pairs is desired to build a complete device with minimal heat losses.

Both n-type and p-type ionogels with high ionic Seebeck coefficients and matched electrical and thermal conductivities are needed when integrating a complete iTE device (*38*). In this regard, p-type and n-type iTE legs derived from the same-parent polymer are the best choices (*8*, *39*) because they have more similarities and are better adapted to the same application environment. To fabricate all-PEO–based iTE legs, we synthesized p-type PEO/EmimCl ionogels. As shown in Fig. 5D and fig. S17, p-type PEO/EmimCl has a decent *S*_i_ of 13.5 mV K^−1^. The ionic electrical conductivity and thermal conductivity of p-type PEO/EmimCl (0.5 mS cm^−1^, 0.29 W m^−1^ K^−1^) and n-type PEO/LiTFSI_50%_-0.6EmimBF_4_ (1.79 mS cm^−1^, 0.11 W·m^−1^ K^−1^) are in the same order of magnitude, representing a good performance matching. As a result, the thermopower of all-PEO–based iTE device is significantly enhanced by connecting multiple p-n pairs (Fig. 5E). For example, a device consisting of three pairs produces a high thermopower of −80 mV K^−1^. In addition, the all-PEO–based iTE device shows good thermal response and excellent repeatability during continuous heat-on and heat-off cycles under different temperature gradients (Fig. 5F). As a showcase for energy harvesting, we attached an all-PEO–based iTE device containing two p-n pairs to a cup containing warm water (Fig. 5G). A thermal voltage of approximately 426 mV is immediately generated under a temperature gradient of about 8.5 K between the warm water and the ambient environment, revealing great potential for applications in low-grade heat harvesting, temperature monitoring, and sensing.

## DISCUSSION

In summary, n-type PEO-based iTE gels with combined advantages of outstanding PF, mechanical adaptability, self-healing, and recyclability are developed. The n-type electrical transport mechanism is investigated in detail by experimentally analyzing the microscopic structures and spectra, in combination with theoretical calculations on items such as diffusion coefficients, Eastman entropy changes, and ionic binding energy. Different from previous studies that use the weak interaction between cations and oxygen to facilitate cation transport, we introduce strong Li^+^-ether oxygen coordination and create anion-rich ion clusters through ion-selective association at high salt concentration, which allows for anion migration–dominated n-type iTE performance. Prototypes consisting of all PEO-based ionogels exhibit high thermopower and rapid response to temperature change. The development of n-type iTE gel with fascinating thermoelectric, mechanical, self-healing, and recyclability behaviors will pave the way for the design of iTE materials with multifunctionality. We believe that our strategy of metal coordination and ion-selective association will enrich in-depth studies on the regulation of ion-polymer and ion-ion interactions for more efficient energy conversion and storage.

## MATERIALS AND METHODS

### Materials

PEO powders (weight molecular of 4,000,000 g mol^−1^), LiTFSI, NaTFSI, KTFSI, and succinonitrile were purchased from Aladdin. The EmimBF_4_ and EmimCl were supplied by Lanzhou Greenchem ILS, LIPC, ACS (Lanzhou China). Anhydrous ethanol was purchased from Sinopharm Chemical Reagent Co. Ltd. All reagents were used as received without any further purification.

### Synthesis of ionogels

The ionogels were prepared by a simple solution process, followed by a casting treatment. First, PEO powders were stirred in anhydrous ethanol at 80°C until completely being dissolved. At the same time, LiTFSI was dissolved in ethanol, followed by adding EmimBF_4_ to obtain transparent salt-mixed solutions. The above two solutions were then mixed and stirred for 10 hours. The ionogel films were prepared by pouring the final solutions into a polytetrafluoroethylene mold and then drying at 50°C overnight. For the binary ionogel PEO/LiTFSI*_x_*, *x* indicates the mass percentage of LiTFSI to the total weight of PEO and LiTFSI, ranging from 16.7 to 50 wt %. For all ternary ionogels, 50% weight percentage of LiTFSI is chosen, and a variable amount of EmimBF_4_ denoted as PEO/LiTFSI50%-*y*EmimBF_4_ is studied, where *y* is the weight ratio of EmimBF_4_ to LiTFSI ranging from 0.1 to 0.8. For comparison, NaTFSI and KTFSI with the same moles were used to substitute LiTFSI. Furthermore, succinonitrile was added to the mixture of PEO, LiTFSI, and EmimBF_4_, for tuning the ionic dynamics.

To synthesize p-type PEO-based ionogels, EmimCl was added into the PEO/ethanol solution as described above. The p-n legs integrated modules were assembled by alternately casting p- and n-type ionogels onto the precasted copper electrodes. Polyimide tapes were applied to the module for encapsulation before measurement.

### Characterizations

Samples for Seebeck coefficient measurements were obtained by drop-casting the ionogels on glass or polyimide substrates coated with Cu electrodes and dried as mentioned above. The ionic Seebeck coefficient was measured by a homemade equipment according to $S_{i}=-\frac{V_{h}-V_{c}}{T_{h}-T_{c}}$. A nanovoltmeter (Keithley 2182A) and two T-type thermocouples connected with Keithley 7710 multimeter were used to collect the open circuit voltages and the temperature gradients between the two copper electrodes,respectively. Thermographs were obtained by an infrared camera (FOTRIC 226). The ionic conductivity σ_i_ was calculated as follows: $\sigma_{i}=\frac{d}{A}\frac{1}{R}$ by sandwiching the ionogel film between two stainless steel plates. The *d*, *A*, and *R* in the formula represent the thickness, area, and ionic resistance, respectively. The ionic resistance was tested by electrochemical impedance spectroscopy on an electrochemical workstation (DH7006,) with the frequency ranging from 0.1 to 105 Hz. The thermal conductivity was measured via a transient heating method through the Thermal Conductivity Analyzer (TC3200). All thermoelectric performance were characterized at room temperature and 50 to 60% RH unless specified.

The crystallinity was analyzed by XRD (Dandong Haoyuan DX-2700B) with a Cu K_α_ radiation source (40 KV, 30 mA). The ultraviolet (UV)–visible transmittance spectra were acquired with a UV-3600 UV spectrophotometer. The angular frequency dependencies of the storage modulus (*G′*) and loss modulus (*G*″) for ionogels were performed using an AR-G2 rheometer (TA Instrument) with the plate-to-plate configuration. The FTIR spectroscopy was recorded using Nicolet6700 with the attenuated total reflection accessory. Raman spectra were obtained on an InVia Reflex Renishaw spectrometer using a 785-nm laser wavelength. Stress-strain curves were performed on an Instron5969 testing instrument with a speed of 10 mm min^−1^ at 25°C. NMR spectra were measured via a Bruker AVANCE400 NMR spectrometer (400 MHz) in acetonitrile-d. Optical microscopy images during the self-repair process were obtained using polarized optical microscopy (Leica DM2500P).

### Computational details

The parameters of the bond, angle, dihedral, van der Waals interactions, and electrostatic interactions of LiTFSI, EmimBF_4_, and PEO (C180H362O90) were described by the all-atom optimized potential for liquid simulations (OPLS-AA) force field, which has been used successfully to determine the structures and properties of mixed systems. The simulated system I contains six PEO chains, each 90 monomers long and terminated with hydrogen, with 83 LiTFSI for the PEO/LiTFSI case (5120 total atoms). Additional 72 EmimBF_4_ were introduced in system II for the PEO/LiTFSI-EmimBF_4_ case (6848 total atoms). The parameters in the OPLS-AA and electric state were established using the PolyParGen software. The nonbonding interactions between distinct atoms include both electrostatic and van der Waals terms. The former one was calculated via the particle-particle-particle-mesh algorithm with an accuracy of 0.0001. The latter one was computed by the 12-6 Lennard-Jones potential, which was truncated at 1.2 nm. The SHAKE algorithm was used to minimize high-frequency vibrations in O─H bonds. The mixing principles of Lorentz-Berthelot are used to model the parameters between different atomic species.

## Supplementary Materials

### This PDF file includes:

Figs. S1 to S17 Table S1 References

## References

[1] C.-G. Han, X. Qian, Q. Li, B. Deng, Y. Zhu, Z. Han, W. Zhang, W. Wang, S.-P. Feng, G. Chen, W. Liu, Giant thermopower of ionic gelatin near room temperature. Science 368, 1091–1098 (2020). 32354840 10.1126/science.aaz5045

[2] B. Kim, J. U. Hwang, E. Kim, Chloride transport in conductive polymer films for an n-type thermoelectric platform. Energ. Environ. Sci. 13, 859–867 (2020).

[3] S. L. Kim, H. T. Lin, C. Yu, Thermally chargeable solid-state supercapacitor. Adv. Energy Mater. 6, 1600546 (2016).

[4] D. Zhao, A. Wurger, X. Crispin, Ionic thermoelectric materials and devices. J. Energy Chem. 61, 88–103 (2021).

[5] Y. Zhou, Z. Dong, Y. He, W. Zhu, Y. Yuan, H. Zeng, C. Li, S. Chen, K. Sun, Multi-ionic hydrogel with outstanding heat-to-electrical performance for low-grade heat harvesting. Chem. Asian J. 17, e202200850 (2022). 36074542 10.1002/asia.202200850

[6] C. Jiang, X. Lai, Z. Wu, H. Li, X. Zeng, Y. Zhao, Q. Zeng, J. Gao, Y. Zhu, A high-thermopower ionic hydrogel for intelligent fire protection. J. Mater. Chem. A 10, 21368–21378 (2022).

[7] Y. Zhang, Y. Dai, F. Xia, X. Zhang, Gelatin/polyacrylamide ionic conductive hydrogel with skin temperature-triggered adhesion for human motion sensing and body heat harvesting. Nano Energy 104, 107977 (2022).

[8] Y.-H. Pai, J. Tang, Y. Zhao, Z. Liang, Ionic organic thermoelectrics with impressively high thermopower for sensitive heat harvesting scenarios. Adv. Energy Mater. 13, 2202507 (2022).

[9] C. Chi, M. An, X. Qi, Y. Li, R. Zhang, G. Liu, C. Lin, H. Huang, H. Dang, B. Demir, Y. Wang, W. Ma, B. Huang, X. Zhang, Selectively tuning ionic thermopower in all-solid-state flexible polymer composites for thermal sensing. Nat. Commun. 13, 221 (2022). 35017492 10.1038/s41467-021-27885-2 PMC8752756

[10] D. Zhao, A. Martinelli, A. Willfahrt, T. Fischer, D. Bernin, Z. U. Khan, M. Shahi, J. Brill, M. P. Jonsson, S. Fabiano, X. Crispin, Polymer gels with tunable ionic Seebeck coefficient for ultra-sensitive printed thermopiles. Nat. Commun. 10, 1093 (2019). 30842422 10.1038/s41467-019-08930-7 PMC6403253

[11] Z. A. Akbar, Y. T. Malik, D.-H. Kim, S. Cho, S.-Y. Jang, J.-W. Jeon, Self-healable and stretchable ionic-liquid-based thermoelectric composites with high ionic Seebeck coefficient. Small 18, e2106937 (2022). 35344267 10.1002/smll.202106937

[12] X. He, H. Cheng, S. Yue, J. Ouyang, Quasi-solid state nanoparticle/(ionic liquid) gels with significantly high ionic thermoelectric properties. J. Mater. Chem. A 8, 10813–10821 (2020).

[13] Z. Liu, H. Cheng, Q. Le, R. Chen, J. Li, J. Ouyang, Giant thermoelectric properties of ionogels with cationic doping. Adv. Energy Mater. 12, 2200858 (2022).

[14] S. Mardi, D. Zhao, N. Kim, I. Petsagkourakis, K. Tybrandt, A. Reale, X. Crispin, The interfacial effect on the open circuit voltage of ionic thermoelectric devices with conducting polymer electrodes. Adv. Electron. Mater. 7, 2100506 (2021).

[15] S. Liu, Y. Yang, H. Huang, J. Zheng, G. Liu; T. H. To, B. Huang, Giant and bidirectionally tunable thermopower in nonaqueous ionogels enabled by selective ion doping. Sci. Adv. 8, eabj3019 (2022). 34985956 10.1126/sciadv.abj3019 PMC8730620

[16] S. Liu, Y. Yang, S. Chen, J. Zheng, D. G. Lee, D. Li, J. Yang, B. Huang, High p- and n-type thermopowers in stretchable self-healing ionogels. Nano Energy 100, 107542 (2022).

[17] J. Xu, H. Wang, X. Du, X. Cheng, Z. Du, H. Wang, Highly stretchable PU ionogels with self-healing capability for a flexible thermoelectric generator. ACS Appl. Mater. Interfaces 13, 20427–20434 (2021). 33882665 10.1021/acsami.1c03328

[18] H. Cheng, X. He, Z. Fan, J. Ouyang, Flexible quasi-solid state ionogels with remarkable Seebeck coefficient and high thermoelectric properties. Adv. Energy Mater. 9, 1901085 (2019).

[19] M. Bonetti, S. Nakamae, M. Roger, P. Guenoun, Huge Seebeck coefficients in nonaqueous electrolytes. J. Chem. Phys. 134, 114513 (2011). 21428638 10.1063/1.3561735

[20] J. Atik, D. Diddens, J. H. Thienenkamp, G. Brunklaus, M. Winter, E. Paillard, Cation-assisted lithium-ion transport for high-performance PEO-based ternary solid polymer electrolytes. Angew. Chem. Int. Ed. 60, 11919–11927 (2021). 10.1002/anie.202016716 PMC8252488 33645903

[21] Y. Su, X. Rong, A. Gao, Y. Liu, J. Li, M. Mao, X. Qi, G. Chai, Q. Zhang, L. Suo, L. Gu, H. Li, X. Huang, L. Chen, B. Liu, Y.-S. Hu, Rational design of a topological polymeric solid electrolyte for high-performance all-solid-state alkali metal batteries. Nat. Commun. 13, 4181 (2022). 35854015 10.1038/s41467-022-31792-5 PMC9296621

[22] P. Kang, L. Wu, D. Chen, Y. Su, Y. Zhu, J. Lan, X. Yang, G. Sui, Dynamical ion association and transport properties in PEO-LiTFSI electrolytes: Effect of salt concentration. J. Phys. Chem. B 126, 4531–4542 (2022). 35695471 10.1021/acs.jpcb.2c01523

[23] D. J. Brooks, B. V. Merinov, W. A. Goddard III, B. Kozinsky, J. Mailoa, Atomistic description of ionic diffusion in PEO-LiTFSI: Effect of temperature, molecular weight, and ionic concentration. Macromolecules 51, 8987–8995 (2018).

[24] N. Molinari, J. P. Mailoa, B. Kozinsky, Effect of salt concentration on ion clustering and transport in polymer solid electrolytes: A molecular dynamics study of PEO-LiTFSI. Chem. Mater. 30, 6298–6306 (2018).

[25] W. Zhao, T. Sun, Y. Zheng, Q. Zhang, A. Huang, L. Wang, W. Jiang, Tailoring intermolecular interactions towards high-performance thermoelectric ionogels at low humidity. Adv. Sci. 9, 2201075 (2022). 10.1002/advs.202201075 PMC9284173 35478492

[26] W. Zhao, Z. Lei, P. Wu, Mechanically adaptative and environmentally stable ionogels for energy harvest. Adv. Sci. 10, 2300253 (2023). 10.1002/advs.202300253 PMC10288276 37083268

[27] W. Zhan, H. Zhang, X. Lyu, Z.-Z. Luo, Y. Yu, Z. Zou, An ultra-tough and super-stretchable ionogel with multi functions towards flexible iontronics. Sci. China Mater. 66, 1539–1550 (2023).

[28] S. J. Wen, T. J. Richardson, D. I. Ghantous, K. A. Striebel, P. N. Ross, E. J. Cairns, FTIR characterization of PEO+LiN(CF_3_SO_2_)_2_ electrolytes. J. Electroanal. Chem. 408, 113–118 (1996).

[29] S. Lascaud, M. Perrier, A. Vallee, S. Besner, J. Prudhomme, M. Armand, Phase diagrams and conductivity behavior of poly(ethylene oxide) molten salt rubbery electrolytes. Macromolecules 27, 7469–7477 (1994).

[30] F. Fu, Y. Zheng, N. Jiang, Y. Liu, C. Sun, A. Zhang, H. Teng, L. Sun, H. Xie, A dual-salt PEO-based polymer electrolyte with cross-linked polymer network for high-voltage lithium metal batteries. Chem. Eng. J. 450, 137776 (2022).

[31] N. R. Dhumal, S. P. Gejji, Theoretical studies on blue versus red shifts in diglyme-M^+^-X^−^ (M = Li, Na, and K and X = CF_3_SO_3_, PF_6_, and (CF_3_SO_2_)_2_N). J. Phys. Chem. A 110, 219–227 (2006). 16392858 10.1021/jp054209g

[32] S. Xu, Z. Sun, C. Sun, F. Li, K. Chen, Z. Zhang, G. Hou, H.-M. Cheng, F. Li, Homogeneous and fast ion conduction of PEO-based solid-state electrolyte at low temperature. Adv. Funct. Mater. 30, 2007172 (2020).

[33] L. Li, W. Li, X. Wang, X. Zou, S. Zheng, Z. Liu, Q. Li, Q. Xia, F. Yan, Ultra-tough and recyclable ionogels constructed by coordinated supramolecular solvents. Angew. Chem. Int. Ed. 61, e202212512 (2022). 10.1002/anie.202212512 36264066

[34] Y. Yu, G. Huang, J.-Y. Du, J.-Z. Wang, Y. Wang, Z.-J. Wu, X.-B. Zhang, A renaissance of N,N-dimethylacetamide-based electrolytes to promote the cycling stability of Li-O_2_ batteries. Energ. Environ. Sci. 13, 3075–3081 (2020).

[35] D.-J. Yoo, Q. Liu, O. Cohen, M. Kim, K. A. A. Persson, Z. Zhang, Rational design of fluorinated electrolytes for low temperature lithium-ion batteries. Adv. Energy Mater. 13, 2204182 (2023).

[36] Z. Cao, H. Liu, L. Jiang, Transparent, mechanically robust, and ultrastable ionogels enabled by hydrogen bonding between elastomers and ionic liquids. Mater. Horiz. 7, 912–918 (2020).

[37] P. Xu, S. Wang, A. Lin, H.-K. Min, Z. Zhou, W. Dou, Y. Sun, X. Huang, H. Tran, X. Liu, Conductive and elastic bottlebrush elastomers for ultrasoft electronics. Nat. Commun. 14, 623–623 (2023). 36739447 10.1038/s41467-023-36214-8 PMC9899285

[38] B. Chen, Q. Chen, S. Xiao, J. Feng, X. Zhang, T. Wang, Giant negative thermopower of ionic hydrogel by synergistic coordination and hydration interactions. Sci. Adv. 7, eabi7233 (2021). 34818039 10.1126/sciadv.abi7233 PMC8612679

[39] M. Jiang, Y. Fu, Q. Zhang, Z. Hu, A. Huang, S. Wang, L. Wang, W. Jiang, High-efficiency and reliable same-parent thermoelectric modules using Mg_3_Sb_2_-based compounds. Natl. Sci. Rev. 10, nwad095 (2023). 37181092 10.1093/nsr/nwad095 PMC10174719

[40] Z. Liu, H. Cheng, H. He, J. Li, J. Ouyang, Significant enhancement in the thermoelectric properties of ionogels through solid network engineering. Adv. Funct. Mater. 32, 2109772 (2022).

[41] D. Song, C. Chi, M. An, Y. Du, W. Ma, K. Wang, X. Zhang, Ionic Seebeck coefficient and figure of merit in ionic thermoelectric materials. Cell Rep. Phys. Sci. 3, 101018 (2022).

