# Supplementary material for: Exceptional n-type thermoelectric ionogels enabled by metal coordination and ion-selective association
Source: Sci Adv. 2023 Oct 25;9(43):eadk2098. doi: 10.1126/sciadv.adk2098 (PMC10599631; doi:10.1126/sciadv.adk2098)
Supplement: Supplementary file 1 — Figs. S1 to S17 Table S1 References [file sciadv.adk2098_sm.pdf]

Science Advances
AAAS

Supplementary Materials for

Exceptional n-type thermoelectric ionogels enabled by metal coordination and ion-selective association

Wei Zhao *et al.*

Corresponding author: Qihao Zhang, qihao.zhang@kit.edu; Lianjun Wang, wanglj@dhu.edu.cn; Wan Jiang, wanjiang@dhu.edu.cn

Sci. Adv. **9**, eadk2098 (2023)
DOI: 10.1126/sciadv.adk2098

This PDF file includes:

Figs. S1 to S17
Table S1
References

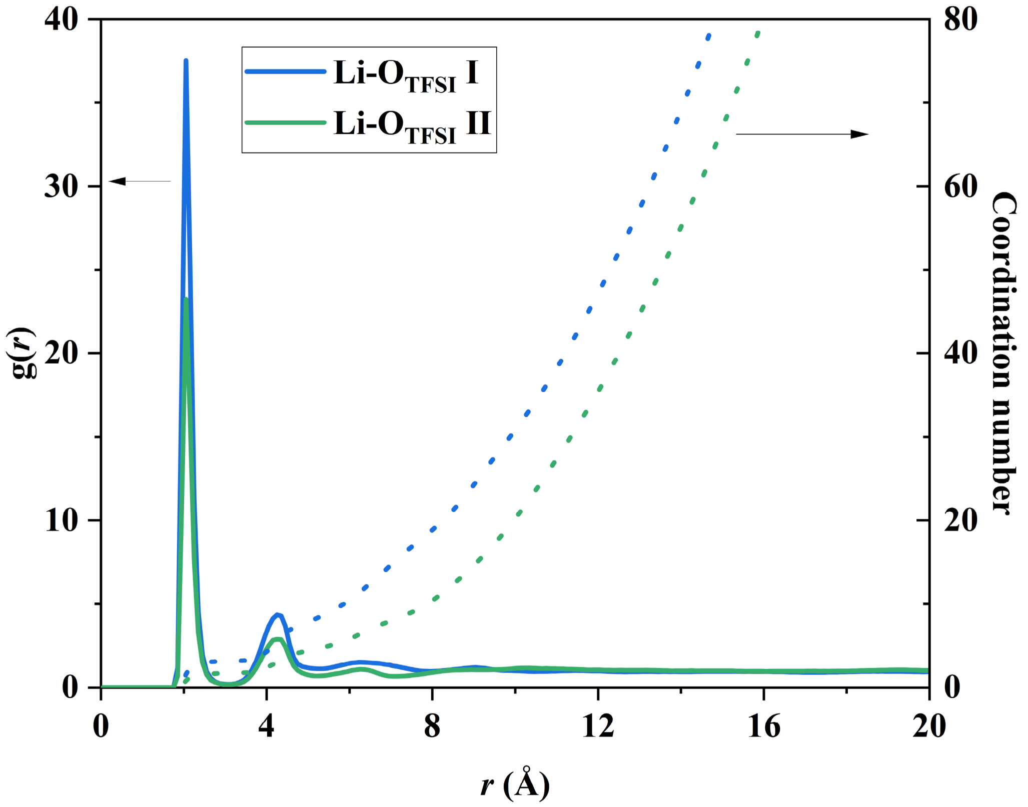

Fig. S1. The radial distribution function and coordination number plots of Li^+-O$_{TFSI}$.

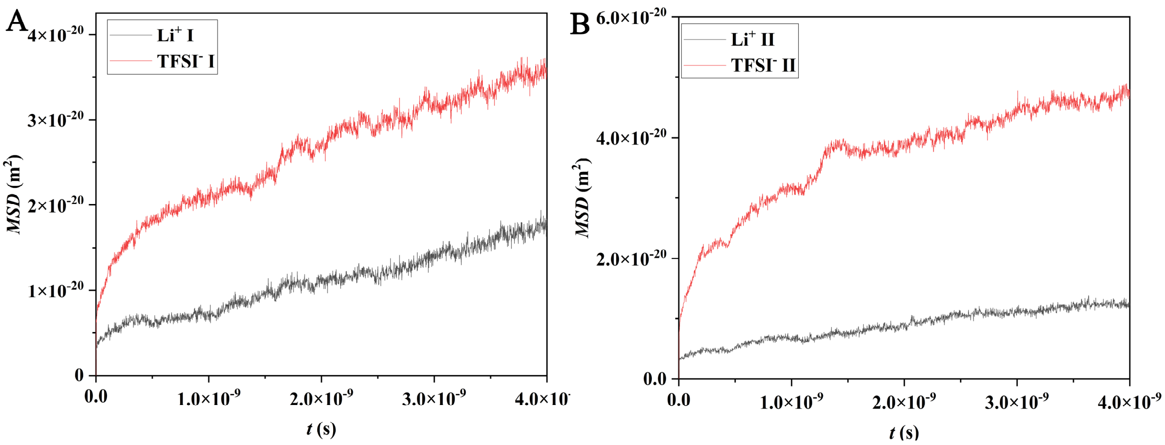

Fig. S2. The mean-squared displacement of anion and cation in A) PEO/LiTFSI and B) PEO/LiTFSI-EmimBF$_4$.

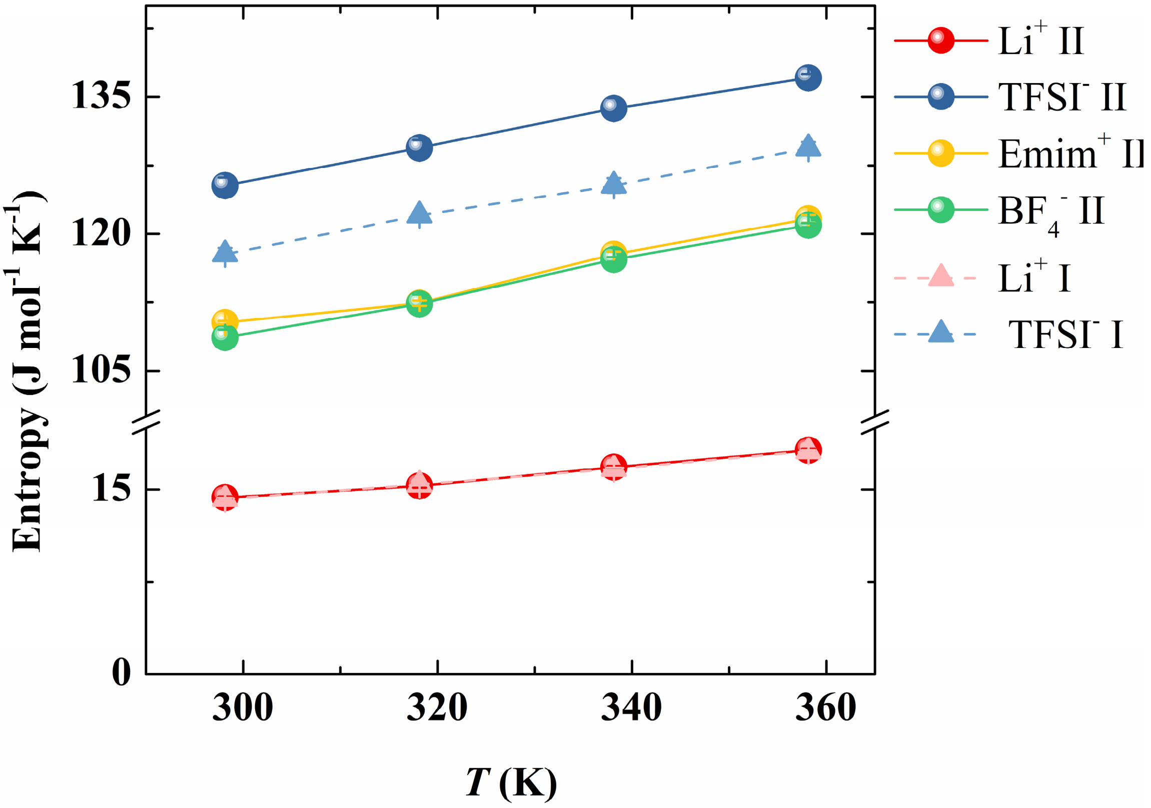

Fig. S3. The entropy of cation and anion in PEO/LiTFSI and PEO/LiTFSI-EmimBF4 with the temperature from 298.15 to 358.15 K.

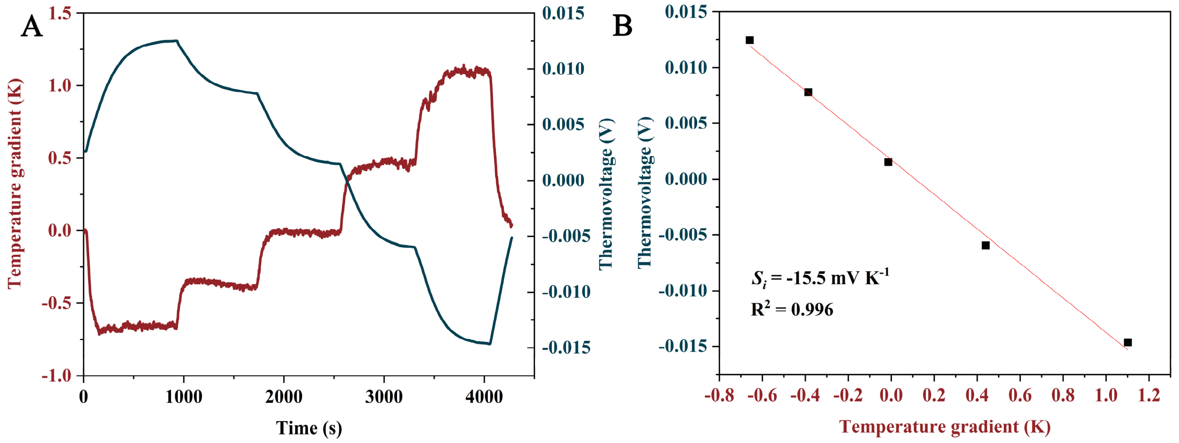

Fig. S4. Thermovoltage of PEO/LiTFSI$_{50\%}$-0.6EmimBF$_4$ as a function of temperature gradient. Variation of thermovoltage with the temperature gradient exhibits a good linear fitting relationship, from which the ionic Seebeck coefficient can be obtained.

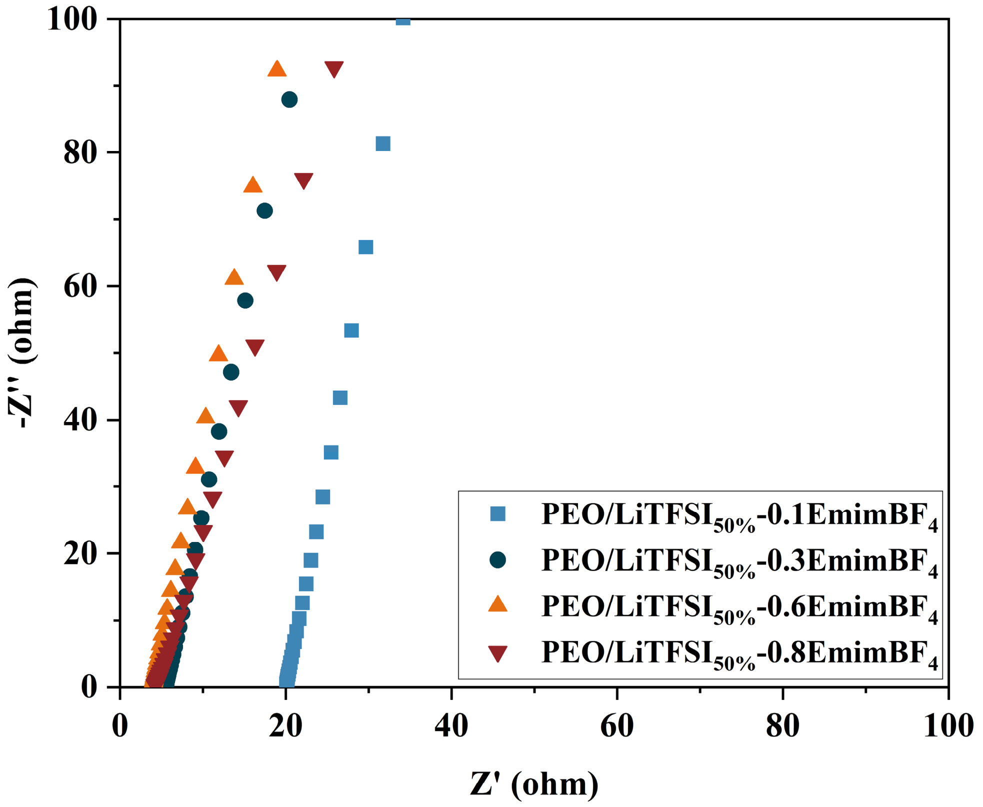

Fig. S5. Nyquist plots of the PEO/$LiTFSI_{50\%}$-y$EmimBF_4$ with different $EmimBF_4$ loadings.

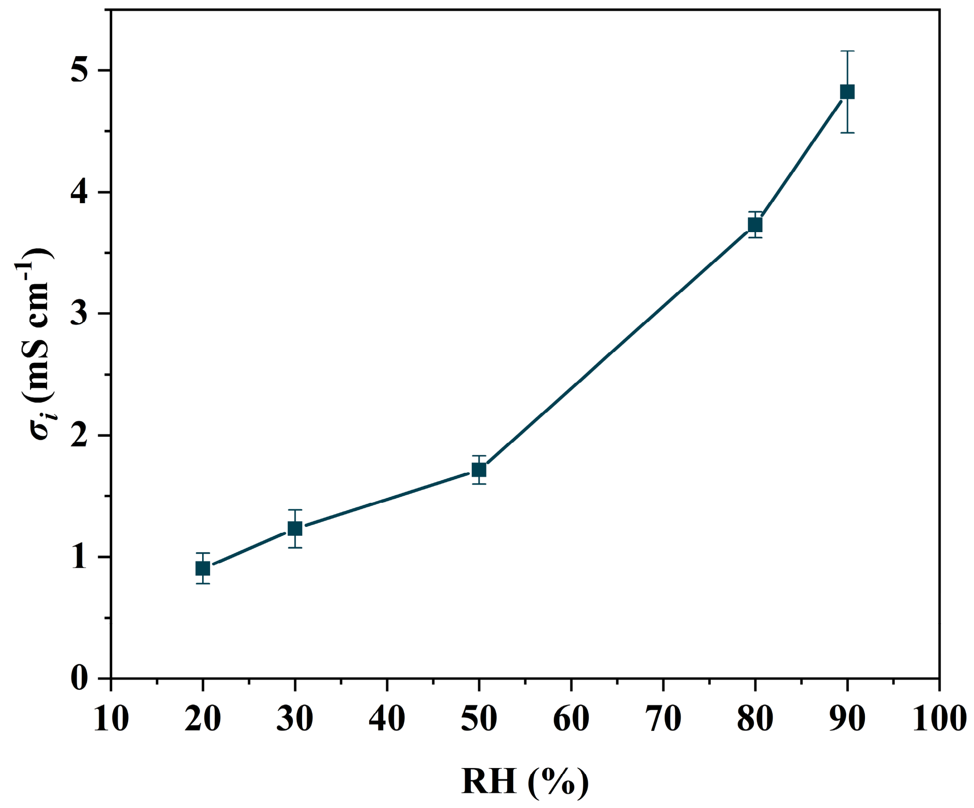

Fig. S6. Ionic conductivity of PEO/LiTFSI$_{50\%}$-0.6EmimBF$_4$ ionogels versus relative humidity.

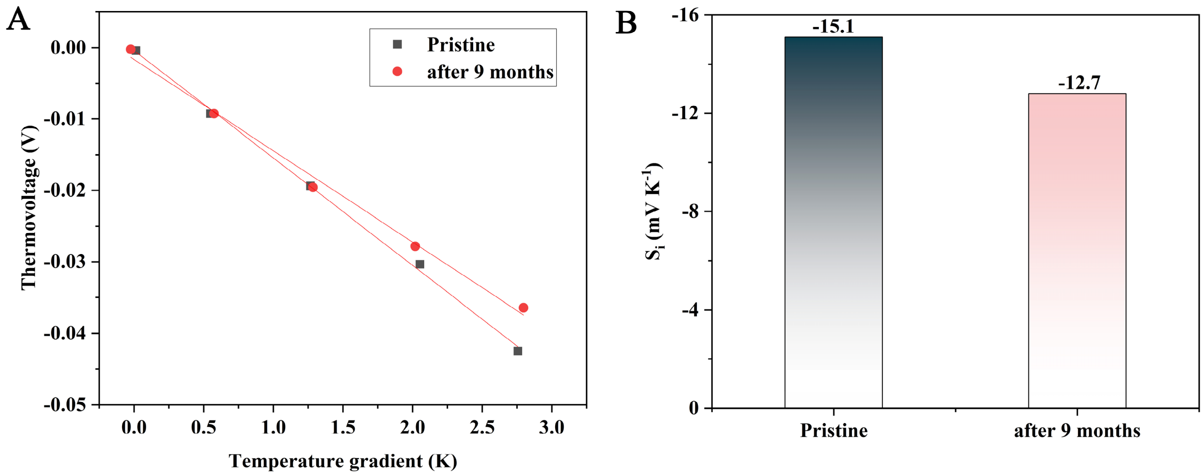

Fig. S7. Thermovoltage of PEO/$LiTFSI_{50\%}$-0.6$EmimBF_4$ before and after placing in vacuum environment for 9 months.

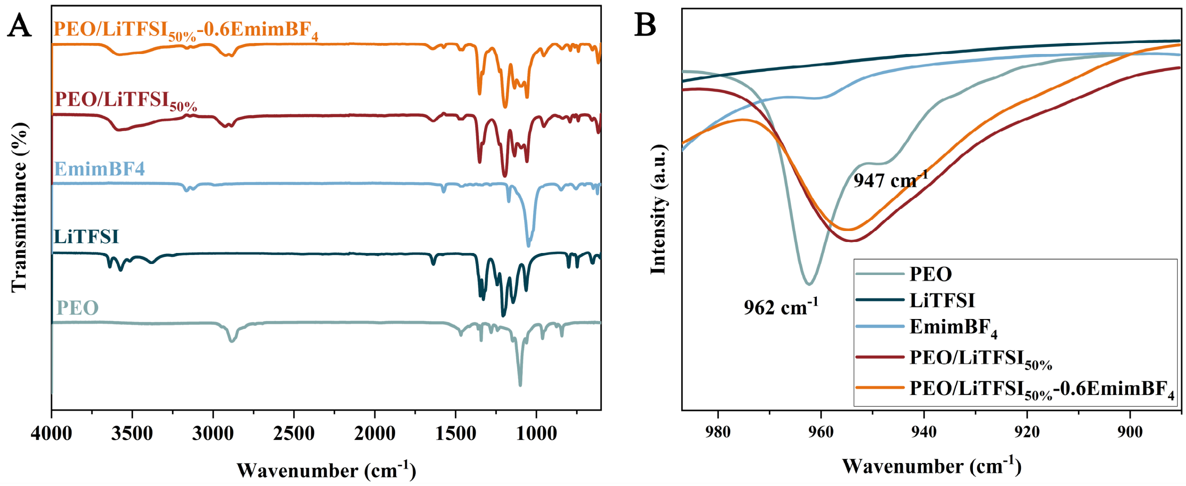

Fig. S8. FTIR spectra of PEO, LiTFSI, $EmimBF_4$, PEO/$LiTFSI_{50\%}$ and PEO/$LiTFSI_{50\%}$-0.6$EmimBF_4$ in the range of A) 4000-600 cm^{-1} and B) 987-890 cm^{-1}.

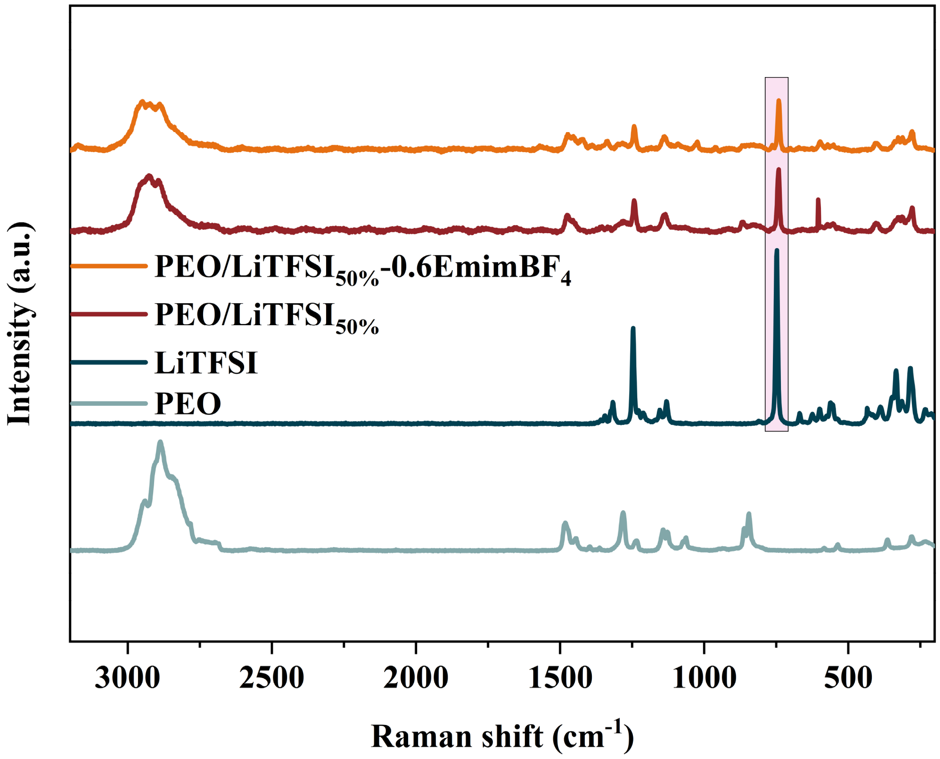

Fig. S9. Raman spectra of PEO, LiTFSI, PEO/$LiTFSI_{50\%}$ and PEO/$LiTFSI_{50\%}$-0.6$EmimBF_4$ in the range of 3200-400 cm^{-1}.

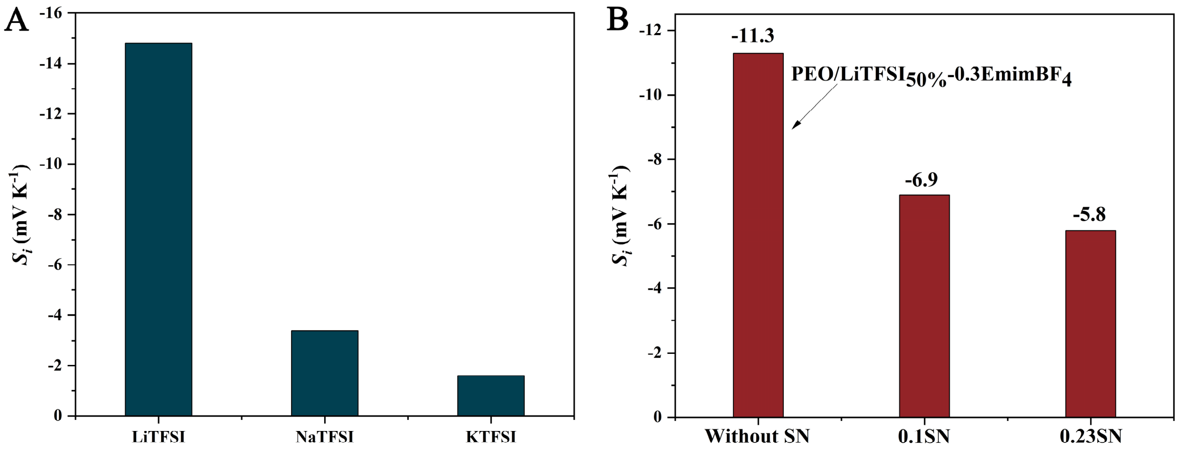

Fig. S10. Ionic Seebeck coefficient of the ionogels. A) With different alkali metal salt and B) the variation after adding different weight of succinonitrile (SN).

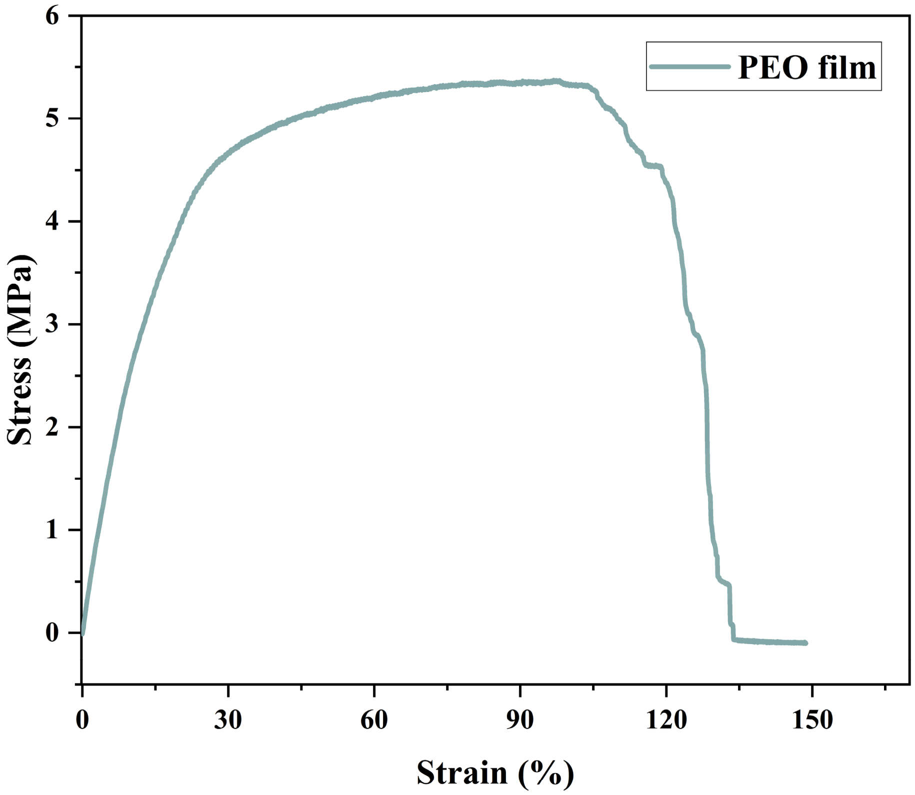

Fig. S11. Tensile stress–strain curves of the PEO film at stretching rate of 10 mm min^{-1}.

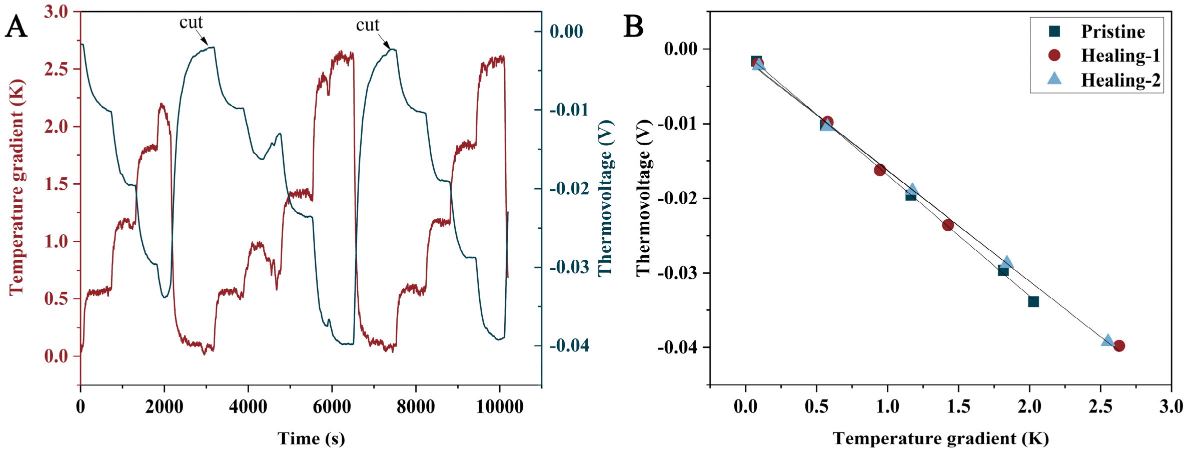

Fig. S12. Thermovoltage of PEO/$LiTFSI_{50\%}$-0.6EmimBF_4 during the repeat cut-healing process.

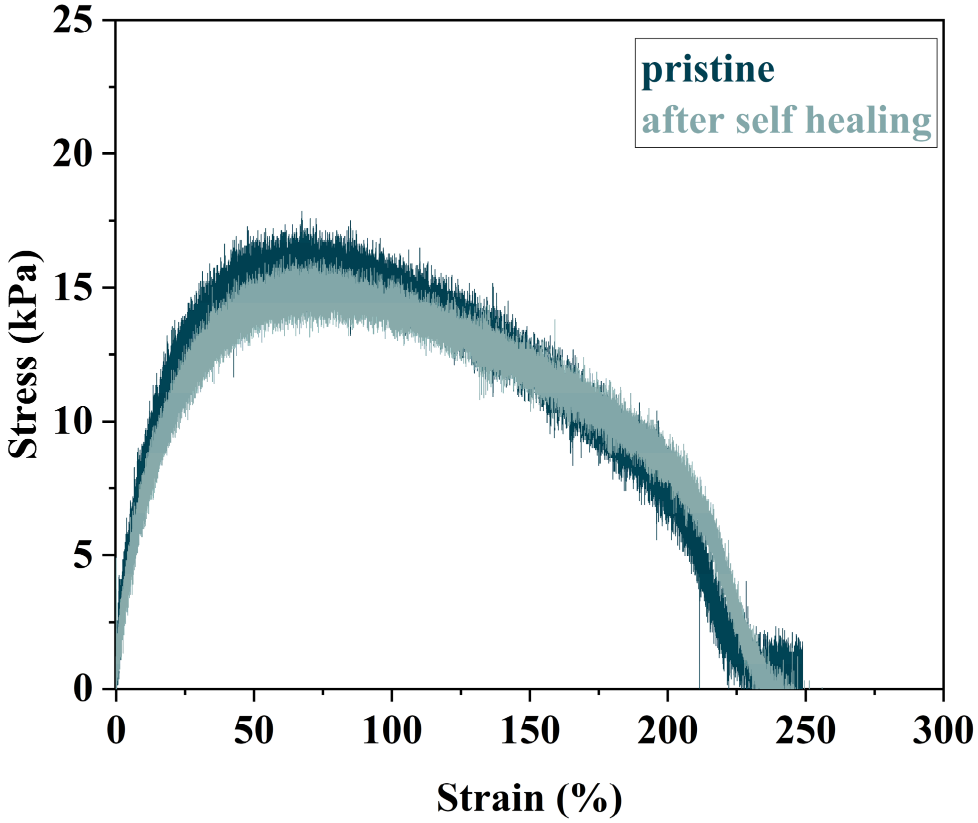

Fig. S13. Tensile strain-stress curves of the pristine and healed samples.

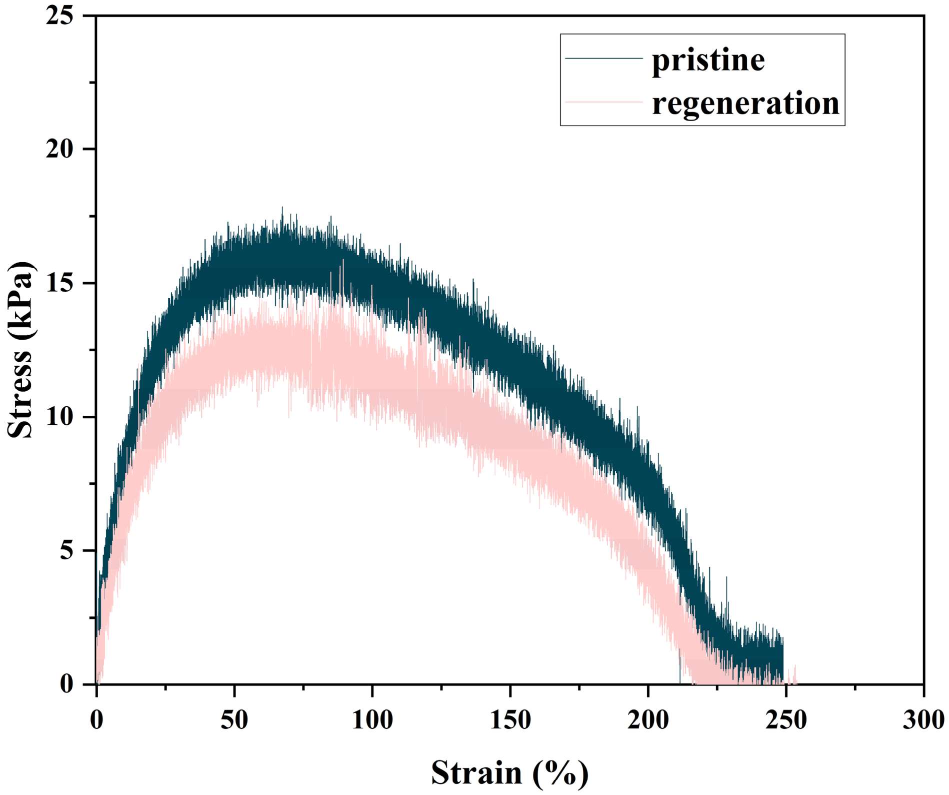

Fig. S14. Typical stress–strain curves of pristine and regenerated samples.

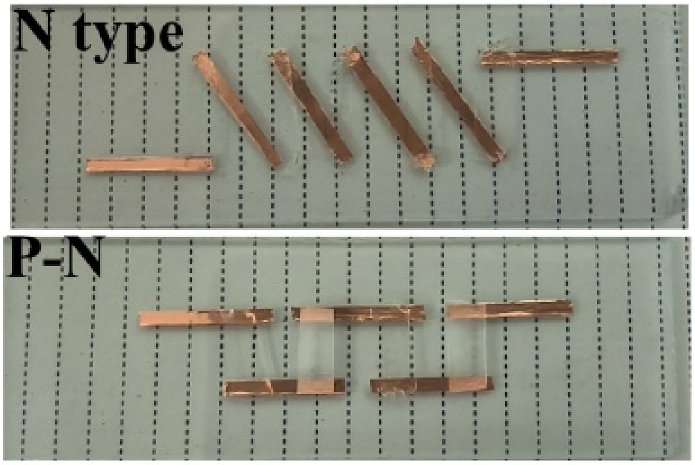

Fig. S15. Digital photos of the assembled in-plane i-TEG with five n-type legs (above) and two pairs of p-n legs (bottom).

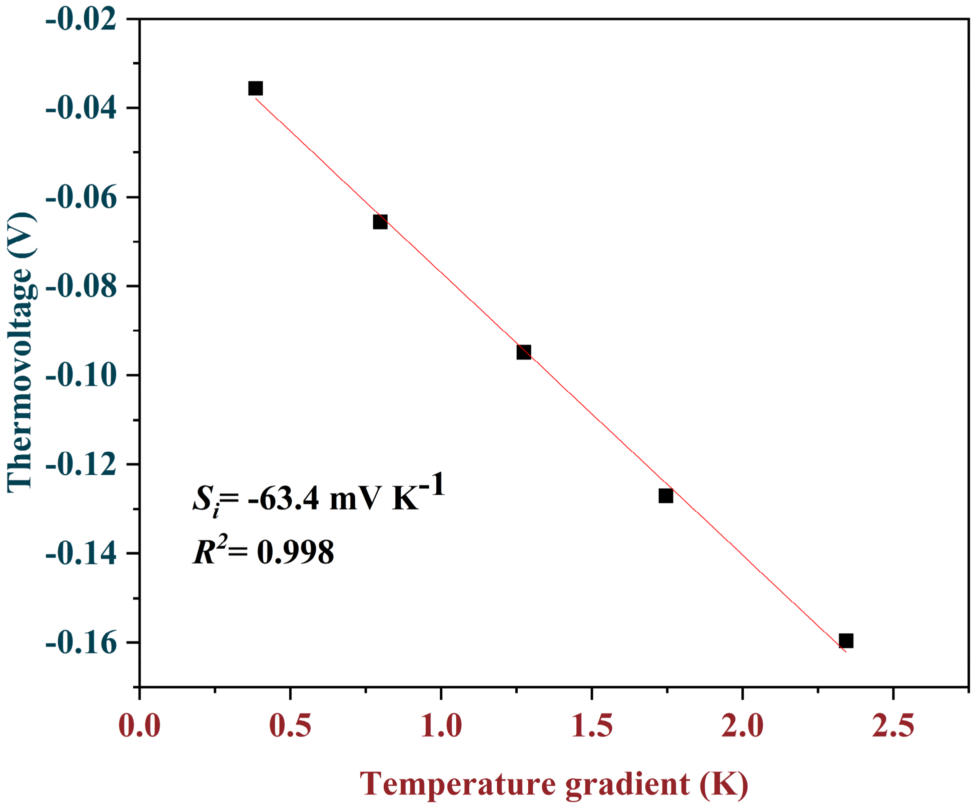

Fig. S16. Thermovoltage as a function of temperature gradient for the assembled n-type device with 5 legs. Variation of thermovoltage with the temperature gradient exhibits a good linear fitting relationship, from which the ionic Seebeck coefficient can be obtained.

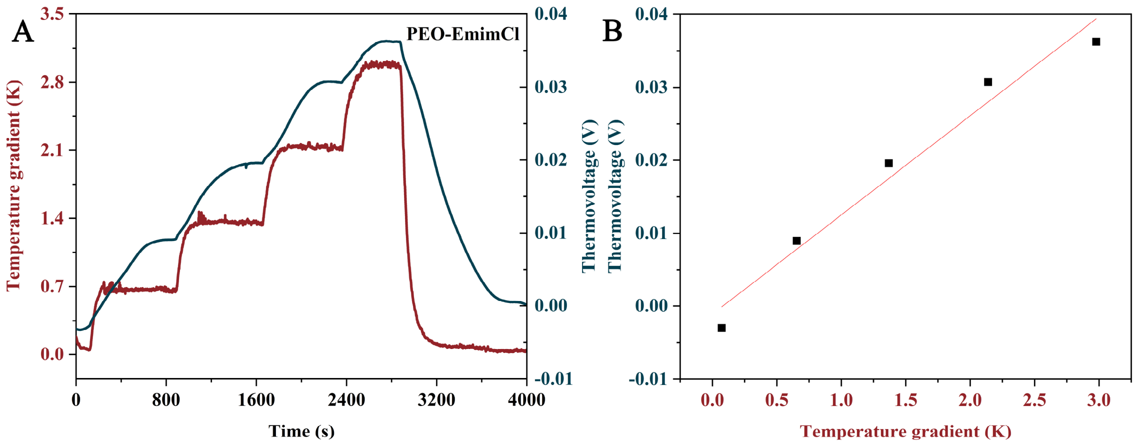

Fig. S17. The generated thermovoltage of p-type PEO-EmimCl leg under different temperature gradient.

Note that the electrical conductivity of iTE materials is still lower than that of electronic thermoelectric materials. In order to further enhance the ionic conductivity, there are some potential strategies. One is to introduce an amorphous structure of polymer matrix, which can facilitate the ionic transport. For example, an antisolvent method has been proposed to engineer the microstructure of ionogels, which increases the ionic conductivity from 7 to 17.6 $mS \cdot cm^{-1}$ (*40*). In addition, based on the Stokes-Einstein equation, ionic mobility is inversely proportional to the viscosity of ionic liquid. The lower viscosity of the ionic liquid, the higher ionic conductivity, so choosing ionic liquid with low viscosity is also favorable to improve the conductivity (*13*). Yet, it is worth mentioning that even though iTE materials would have higher zT_i, it is impractical to make a direct comparison between zT_i and electronic zT (*41*). This is because they have different modes of operation. Ionogels cannot be used as thermoelectric generators because the ions cannot pass through the electrodes into the external circuit. Instead, they can be used in ionic thermoelectric capacitors (*41*).

Table S1. Detailed information about the calculated RDF and CN for Li-O_{TFSI} (Left) and Li-O_{PEO} (Right).

System		*r* (Å)	*g*(*r*)	*CN*
I	Peak	**2.050**	**37.532**	1.582
	Valley	3.150	0.187	**3.156**
II	Peak	**2.050**	**23.241**	0.804
	Valley	3.150	0.176	**1.703**

System		*r* (Å)	*g*(*r*)	*CN*
I	Peak	**2.050**	**14.275**	1.118
	Valley	3.250	0.156	**2.261**
II	Peak	**2.050**	**16.054**	1.018
	Valley	3.250	0.156	**2.173**

REFERENCES AND NOTES

1. C.-G. Han, X. Qian, Q. Li, B. Deng, Y. Zhu, Z. Han, W. Zhang, W. Wang, S.-P. Feng, G. Chen, W. Liu, Giant thermopower of ionic gelatin near room temperature. *Science* **368**, 1091–1098 (2020).

2. B. Kim, J. U. Hwang, E. Kim, Chloride transport in conductive polymer films for an n-type thermoelectric platform. *Energ. Environ. Sci.* **13**, 859–867 (2020).

3. S. L. Kim, H. T. Lin, C. Yu, Thermally chargeable solid-state supercapacitor. *Adv. Energy Mater.* **6**, 1600546 (2016).

4. D. Zhao, A. Wurger, X. Crispin, Ionic thermoelectric materials and devices. *J. Energy Chem.* **61**, 88–103 (2021).

5. Y. Zhou, Z. Dong, Y. He, W. Zhu, Y. Yuan, H. Zeng, C. Li, S. Chen, K. Sun, Multi-ionic hydrogel with outstanding heat-to-electrical performance for low-grade heat harvesting. *Chem. Asian J.* **17**, e202200850 (2022).

6. C. Jiang, X. Lai, Z. Wu, H. Li, X. Zeng, Y. Zhao, Q. Zeng, J. Gao, Y. Zhu, A high-thermopower ionic hydrogel for intelligent fire protection. *J. Mater. Chem. A* **10**, 21368–21378 (2022).

7. Y. Zhang, Y. Dai, F. Xia, X. Zhang, Gelatin/polyacrylamide ionic conductive hydrogel with skin temperature-triggered adhesion for human motion sensing and body heat harvesting. *Nano Energy* **104**, 107977 (2022).

8. Y.-H. Pai, J. Tang, Y. Zhao, Z. Liang, Ionic organic thermoelectrics with impressively high thermopower for sensitive heat harvesting scenarios. *Adv. Energy Mater.* **13**, 2202507 (2022).

9. C. Chi, M. An, X. Qi, Y. Li, R. Zhang, G. Liu, C. Lin, H. Huang, H. Dang, B. Demir, Y. Wang, W. Ma, B. Huang, X. Zhang, Selectively tuning ionic thermopower in all-solid-state flexible polymer composites for thermal sensing. *Nat. Commun.* **13**, 221 (2022).

10. D. Zhao, A. Martinelli, A. Willfahrt, T. Fischer, D. Bernin, Z. U. Khan, M. Shahi, J. Brill, M. P. Jonsson, S. Fabiano, X. Crispin, Polymer gels with tunable ionic Seebeck coefficient for ultra-sensitive printed thermopiles. *Nat. Commun.* **10**, 1093 (2019).

11. Z. A. Akbar, Y. T. Malik, D.-H. Kim, S. Cho, S.-Y. Jang, J.-W. Jeon, Self-healable and stretchable ionic-liquid-based thermoelectric composites with high ionic Seebeck coefficient. *Small* **18**, e2106937 (2022).

12. X. He, H. Cheng, S. Yue, J. Ouyang, Quasi-solid state nanoparticle/(ionic liquid) gels with significantly high ionic thermoelectric properties. *J. Mater. Chem. A* **8**, 10813–10821 (2020).

13. Z. Liu, H. Cheng, Q. Le, R. Chen, J. Li, J. Ouyang, Giant thermoelectric properties of ionogels with cationic doping. *Adv. Energy Mater.* **12**, 2200858 (2022).

14. S. Mardi, D. Zhao, N. Kim, I. Petsagkourakis, K. Tybrandt, A. Reale, X. Crispin, The interfacial effect on the open circuit voltage of ionic thermoelectric devices with conducting polymer electrodes. *Adv. Electron. Mater.* **7**, 2100506 (2021).

15. S. Liu, Y. Yang, H. Huang, J. Zheng, G. Liu, T. H. To, B. Huang, Giant and bidirectionally tunable thermopower in nonaqueous ionogels enabled by selective ion doping. *Sci. Adv.* **8**, eabj3019 (2022).

16. S. Liu, Y. Yang, S. Chen, J. Zheng, D. G. Lee, D. Li, J. Yang, B. Huang, High p- and n-type thermopowers in stretchable self-healing ionogels. *Nano Energy* **100**, 107542 (2022).

17. J. Xu, H. Wang, X. Du, X. Cheng, Z. Du, H. Wang, Highly stretchable PU ionogels with self-healing capability for a flexible thermoelectric generator. *ACS Appl. Mater. Interfaces* **13**, 20427–20434 (2021).

18. H. Cheng, X. He, Z. Fan, J. Ouyang, Flexible quasi-solid state ionogels with remarkable Seebeck coefficient and high thermoelectric properties. *Adv. Energy Mater.* **9**, 1901085 (2019).

19. M. Bonetti, S. Nakamae, M. Roger, P. Guenoun, Huge Seebeck coefficients in nonaqueous electrolytes. *J. Chem. Phys.* **134**, 114513 (2011).

20. J. Atik, D. Diddens, J. H. Thienenkamp, G. Brunklaus, M. Winter, E. Paillard, Cation-assisted lithium-ion transport for high-performance PEO-based ternary solid polymer electrolytes. *Angew. Chem. Int. Ed.* **60**, 11919–11927 (2021).

21. Y. Su, X. Rong, A. Gao, Y. Liu, J. Li, M. Mao, X. Qi, G. Chai, Q. Zhang, L. Suo, L. Gu, H. Li, X. Huang, L. Chen, B. Liu, Y.-S. Hu, Rational design of a topological polymeric solid electrolyte for high-performance all-solid-state alkali metal batteries. *Nat. Commun.* **13**, 4181 (2022).

22. P. Kang, L. Wu, D. Chen, Y. Su, Y. Zhu, J. Lan, X. Yang, G. Sui, Dynamical ion association and transport properties in PEO-LiTFSI electrolytes: Effect of salt concentration. *J. Phys. Chem. B* **126**, 4531–4542 (2022).

23. D. J. Brooks, B. V. Merinov, W. A. Goddard III, B. Kozinsky, J. Mailoa, Atomistic description of ionic diffusion in PEO-LiTFSI: Effect of temperature, molecular weight, and ionic concentration. *Macromolecules* **51**, 8987–8995 (2018).

24. N. Molinari, J. P. Mailoa, B. Kozinsky, Effect of salt concentration on ion clustering and transport in polymer solid electrolytes: A molecular dynamics study of PEO-LiTFSI. *Chem. Mater.* **30**, 6298–6306 (2018).

25. W. Zhao, T. Sun, Y. Zheng, Q. Zhang, A. Huang, L. Wang, W. Jiang, Tailoring intermolecular interactions towards high-performance thermoelectric ionogels at low humidity. *Adv. Sci.* **9**, 2201075 (2022).

26. W. Zhao, Z. Lei, P. Wu, Mechanically adaptative and environmentally stable ionogels for energy harvest. *Adv. Sci.* **10**, 2300253 (2023).

27. W. Zhan, H. Zhang, X. Lyu, Z.-Z. Luo, Y. Yu, Z. Zou, An ultra-tough and super-stretchable ionogel with multi functions towards flexible iontronics. *Sci. China Mater.* **66**, 1539–1550 (2023).

28. S. J. Wen, T. J. Richardson, D. I. Ghantous, K. A. Striebel, P. N. Ross, E. J. Cairns, FTIR characterization of PEO+$LiN(CF_3SO_2)_2$ electrolytes. *J. Electroanal. Chem.* **408**, 113–118 (1996).

29. S. Lascaud, M. Perrier, A. Vallee, S. Besner, J. Prudhomme, M. Armand, Phase diagrams and conductivity behavior of poly(ethylene oxide) molten salt rubbery electrolytes. *Macromolecules* **27**, 7469–7477 (1994).

30. F. Fu, Y. Zheng, N. Jiang, Y. Liu, C. Sun, A. Zhang, H. Teng, L. Sun, H. Xie, A dual-salt PEO-based polymer electrolyte with cross-linked polymer network for high-voltage lithium metal batteries. *Chem. Eng. J.* **450**, 137776 (2022).

31. N. R. Dhumal, S. P. Gejji, Theoretical studies on blue versus red shifts in diglyme-M^+-X^- (M = Li, Na, and K and X = CF_3SO_3, PF_6, and $(CF_3SO_2)_2N$). *J. Phys. Chem. A* **110**, 219–227 (2006).

32. S. Xu, Z. Sun, C. Sun, F. Li, K. Chen, Z. Zhang, G. Hou, H.-M. Cheng, F. Li, Homogeneous and fast ion conduction of PEO-based solid-state electrolyte at low temperature. *Adv. Funct. Mater.* **30**, 2007172 (2020).

33. L. Li, W. Li, X. Wang, X. Zou, S. Zheng, Z. Liu, Q. Li, Q. Xia, F. Yan, Ultra-tough and recyclable ionogels constructed by coordinated supramolecular solvents. *Angew. Chem. Int. Ed.* **61**, e202212512 (2022).

34. Y. Yu, G. Huang, J.-Y. Du, J.-Z. Wang, Y. Wang, Z.-J. Wu, X.-B. Zhang, A renaissance of N,N-dimethylacetamide-based electrolytes to promote the cycling stability of Li-O_2 batteries. *Energ. Environ. Sci.* **13**, 3075–3081 (2020).

35. D.-J. Yoo, Q. Liu, O. Cohen, M. Kim, K. A. A. Persson, Z. Zhang, Rational design of fluorinated electrolytes for low temperature lithium-ion batteries. *Adv. Energy Mater.* **13**, 2204182 (2023).

36. Z. Cao, H. Liu, L. Jiang, Transparent, mechanically robust, and ultrastable ionogels enabled by hydrogen bonding between elastomers and ionic liquids. *Mater. Horiz.* **7**, 912–918 (2020).

37. P. Xu, S. Wang, A. Lin, H.-K. Min, Z. Zhou, W. Dou, Y. Sun, X. Huang, H. Tran, X. Liu, Conductive and elastic bottlebrush elastomers for ultrasoft electronics. *Nat. Commun.* **14**, 623–623 (2023).

38. B. Chen, Q. Chen, S. Xiao, J. Feng, X. Zhang, T. Wang, Giant negative thermopower of ionic hydrogel by synergistic coordination and hydration interactions. *Sci. Adv.* **7**, eabi7233 (2021).

39. M. Jiang, Y. Fu, Q. Zhang, Z. Hu, A. Huang, S. Wang, L. Wang, W. Jiang, High-efficiency and reliable same-parent thermoelectric modules using Mg_3Sb_2-based compounds. *Natl. Sci. Rev.* **10**, nwad095 (2023).

40. Z. Liu, H. Cheng, H. He, J. Li, J. Ouyang, Significant enhancement in the thermoelectric properties of ionogels through solid network engineering. *Adv. Funct. Mater.* **32**, 2109772 (2022).

41. D. Song, C. Chi, M. An, Y. Du, W. Ma, K. Wang, X. Zhang, Ionic Seebeck coefficient and figure of merit in ionic thermoelectric materials. *Cell Rep. Phys. Sci.* **3**, 101018 (2022).
